# Social cognition remediation interventions: A systematic mapping review

**DOI:** 10.1371/journal.pone.0218720

**Published:** 2019-06-26

**Authors:** Patricia Fernández-Sotos, Iosune Torio, Antonio Fernández-Caballero, Elena Navarro, Pascual González, Mónica Dompablo, Roberto Rodriguez-Jimenez

**Affiliations:** 1 Department of Psychiatry, Instituto de Investigación Sanitaria Hospital 12 de Octubre (imas12), Madrid, Spain; 2 CIBERSAM (Biomedical Research Networking Centre in Mental Health), Madrid, Spain; 3 Universidad Rey Juan Carlos, Madrid, Spain; 4 Instituto de Investigación en Informática de Albacete, Albacete, Spain; 5 Departamento de Sistemas Informáticos, Universidad de Castilla-La Mancha, Albacete, Spain; 6 Cardenal Cisneros, Centro de Enseñanza Superior adscrito a la Universidad Complutense de Madrid, Madrid, Spain; 7 CogPsy-Group, Universidad Complutense de Madrid (UCM), Madrid, Spain; University of Wyoming College of Health Sciences, UNITED STATES

## Abstract

**Background:**

Impairments in social cognition have been described in several psychiatric and neurological disorders. Given the importance of the relationship between social cognition and functioning and quality of life in these disorders, there is a growing interest in social cognition remediation interventions. The aim of this study was to carry out a systematic mapping review to describe the state of the art in social cognition training and remediation interventions.

**Methods:**

Publications from 2006 to 2016 on social cognition interventions were reviewed in four databases: Scopus, PsycINFO, PubMed and Embase. From the initial result set of 3229 publications, a final total of 241 publications were selected.

**Results:**

The study revealed an increasing interest in social cognition remediation interventions, especially in the fields of psychiatry and psychology, with a gradual growth in the number of publications. These were frequently published in high impact factor journals and underpinned by robust scientific evidence. Most studies were conducted on schizophrenia, followed by autism spectrum disorders. Theory of mind and emotional processing were the focus of most interventions, whilst a limited number of studies addressed attributional bias and social perception. Targeted interventions in social cognition were the most frequent practice in the selected papers, followed by non-specific treatment interventions and broad-based interventions.

**Conclusions:**

Research in social cognition remediation interventions is growing. Further studies are needed on attributional bias and social perception remediation programs, while the comparative efficacy of different interventions also remains unclear.

## Introduction

Social cognition refers to the mental operations involved in social interactions, including processes of perceiving, interpreting, and generating responses to the intentions, dispositions, and behaviors of others [1–6]. Social cognition is a key determinant of the proper functioning and development of human beings. Although various disorders, such as autism [7–9], affective disorders [10–13] and eating disorders [14], have been associated with deficits in social cognition, schizophrenia has frequently been the focus of investigation in social cognition [15–17]. The fact that schizophrenia acquired an early paradigmatic position in psychiatry might partly explain this. Nevertheless, some pragmatic reasons may also be identified: (a) A significant number of patients with schizophrenia present deficits in quality of life and functioning; (b) There is an early onset of these deficits and they persist throughout life; (c) The personal, familiar and economic burden of this disorder is very high. (d) Social cognition impairments appear to make an important contribution to deficits in quality of life and functioning [7,18–21].

The last decade has seen a significant increase in research on social cognition and the strategies for its pharmacological and psychosocial treatment. A recent systematic mapping review of pharmacological interventions in social cognition deficits [22] shows an increase in the number of publications, with a significant number studying hypothalamic hormones (especially oxytocin), with a much less frequent focus on antipsychotics, amphetamines, and sex hormones, in that order.

Several non-pharmacological approaches designed to enhance social cognition abilities have also been developed. Some treatment interventions approach social cognition as a unitary construct, but emphasis has recently been placed on a multidimensional structure of social cognition. Initiatives such as the *Social Cognition Psychometric Evaluation* (SCOPE) study have defined social cognition as a multidimensional construct that includes four core domains: emotional processing, social perception, attributional style/bias, and theory of mind [23–25]. In this sense, specific remediation interventions have been developed to improve each of the domains of social cognition, either as the main intervention of the program, or as part of a broader training program targeting a variety of dimensions of the illness. Finally, other psychosocial interventions which do not specifically target social cognition, such as social skills training [26–27], have been shown to have a positive impact on social cognition.

Although several reviews have focused on the usefulness of different social cognition remediation interventions [28–31], to our knowledge, no prior systematic mapping review has examined social cognition remediation programs. A systematic mapping review is a secondary empirical study that provides an overview of the state of the art in a field, identifying venues and topics addressed in the literature. Furthermore, this type of review is a systematic approach to understanding the “map” of a field of knowledge, research question, or practice [32], by identifying linkages rather than results [33]. The presence of this type of study in different fields of medicine has increased, especially in recent years, although systematic mapping reviews are still scarce in psychiatry [34–38].

The aim of the present study was to describe the state of the art in social cognition remediation interventions following a systematic mapping review approach, to provide researchers and clinicians with a global picture on social cognition remediation interventions for psychiatric and other medical conditions.

## Methods

### Research methodology

#### Research interest

The present mapping review has been conducted for several reasons. The first is to perform a broad analysis of the literature on social cognition remediation interventions, with the aim of providing an overview of existing proposals according to a classification criterion. The second is, if possible, to identify existing research gaps in social cognition rehabilitation intervention programs to plan future research, while the third is to identify topics and areas for future systematic literature reviews focused on smaller research areas.

The steps described in the *Template for a Mapping Study Protocol* [39–40] were followed to conduct this study. A mapping review protocol includes three distinct phases: (a) *Research directives* defines the study protocol and identifies the dimensions to be analyzed and the research questions to be answered. (b) *Data collection* gathers relevant papers in accordance with the inclusion and exclusion criteria defined in the protocol. Finally, (c) *Results* maps the existing literature in line with the defined criteria and answers the research questions. Note that the PRISMA checklist [41] has not been used in this paper as it does not in fact fit recommendations for systematic mapping studies. Therefore, a checklist for the *Template for a Mapping Study Protocol* is provided in S1 Table (see supplementary material online).

#### Research directives

In this section, the definition of the research protocol and the description of the research questions is presented. The protocol included the study topic (social cognition remediation interventions); its rationale (previously mentioned); preliminary research questions; and search strategy, selection criteria, and data extraction form. Finally, the protocol included an overview of selected papers in terms of their publication venues and years.

The four research questions (RQ) for this systematic mapping review and their rationale were:

**RQ1. *How many papers have been published on social cognition remediation interventions*? *Is there any temporal trend*? *What is their level of scientific evidence*?**

As previously discussed, deficits in social cognition are commonly found in psychiatric disorders such as autism, depressive disorder, bipolar disorder, and schizophrenia. Due to the prevalence and/or severity of these disorders, and the limited efficacy of pharmacological interventions [42–43], the number of papers published on non-pharmacological social cognition remediation interventions was studied. Second, the possible presence of a temporal trend (increasing or decreasing) was analyzed. Finally, the study of their level of scientific evidence provided complementary information regarding the quality of published studies.

**RQ2. *In what areas of medical knowledge and related disciplines has research on social cognition remediation programs been conducted*?**

Deficits in social cognition are not exclusive to psychiatric disorders. Several neurological disorders such as behavioral-variant frontotemporal dementia [44], traumatic brain injury [45], or Parkinson’s disease [46] are characterized by impairments in social cognition [47]. Other disorders with central nervous system involvement such as tuberous sclerosis [48], fragile X syndrome [49], Prader Willi syndrome [50], or Wilson’s disease [51] have also been related to social cognition impairments. Therefore, it seems interesting to study which areas of knowledge are developing research on social cognition remediation, including medicine and related fields such as psychology and neuroscience.

**RQ3. *Which domains of social cognition are the focus of research in social cognition remediation*?**

Social cognition is considered a multidimensional construct. There is some consensus regarding critical domains of social cognition as four partially overlapping domains (theory of mind/mental state attribution, social perception, attributional bias/style, and emotional processing) [24–25]:

- *Theory of mind* is defined as the ability to recognize intentions, dispositions, and beliefs in oneself and others [52]. Theory of mind includes understanding special ways of communication, such as hints, deception, metaphors or irony, by making inferences about the feelings of others, beliefs, and intentions, and/or by representing human mental states [53–54]. Theory of mind is also known as mentalizing, mental state attribution, or cognitive empathy [55].
- *Social perception* consists of the ability to identify social rules, roles, and goals [56–57]. Social perception is also defined as the ability to correctly interpret the behaviors of other people by means of context and social information [58–59].
- *Attributional bias/style* describes the way in which individuals explain or interpret different social facts or infer the causes of social events [3]. Attributions are external (to others) or internal (to oneself) and may be categorized further by dimensions such as ambiguity of the context or positive vs. negative outcome.
- *Emotional processing* refers to the ability to perceive, recognize and manage emotional information [3]. Emotional processing is also defined as the ability to identify, facilitate, regulate, understand and manage emotions [60–61].

Interventions on social cognition remediation may be focused on one or several social cognition domains. Thus, identifying the most frequently studied domains will help researchers recognize the domains that might need further investigation.

**RQ4. *What types of interventions are employed in social cognitive remediation*?**

Although various forms of interventions may potentially improve social cognition, three main types of training and remediation interventions have been distinguished [31]:

- *Targeted interventions* specifically directed at improving one or more domains of social cognition. Targeted interventions are often subcategorized, including “targeted” and “comprehensive” interventions. “Targeted” refers to brief interventions for a single aspect of social cognition. “Comprehensive” refers to programs that leverage all the elements of an extended training program in order to improve multiple domains of social cognition, typically including practice for the generalization of acquired skills for everyday life. Notice that the present work does not provide this subclassification.
- *Broad-based interventions*, including social cognition training in a broader approach oriented towards improving social cognition, neurocognition and/or social skills.
- *Non-specific interventions*, which do not target social cognition but may produce a positive effect on one or more of its domains.

Classifying published papers according to three main types of interventions facilitates an overview of the specificity of the interventions in social cognition remediation.

### Data collection

With the aim of including relevant and excluding irrelevant papers, the search strategy of this study involved querying reference databases with customized search strings, followed by manual filtering of the query results using predefined inclusion and exclusion criteria. Five researchers were involved in executing the search strategy.

#### Source selection and search string

To minimize the risk of missing relevant papers on social cognition remediation, four reference databases were queried: Scopus (Elsevier), PsycINFO (American Psychological Association), PubMed (American Psychological Association) and Embase (Elsevier).

The keywords for the search string were identified by following mainly research questions RQ3 and RQ4. The first part of the search string was composed of words related to social cognition, using the nomenclature described under RQ3, while the second part included remediation and its synonyms (rehabilitation, therapy, training, treatment and enhancement) related to the types of interventions deduced from RQ4. Finally, all items in our search string were interleaved with or/and statements, to make sure that all relevant papers were retrieved. The final search string was: ("affect perception" OR "affect recognition" OR “attributional bias” OR "attributional style" OR “emotional processing” OR "emotion recognition" OR "mentalizing" OR "theory of mind" OR "social cognition" OR “social perception”) AND (“rehabilitation” OR “remediation” OR “therapy” OR “treatment” OR “training” OR “enhancement”). It should be highlighted that no truncation was used in the terms of the final search string for the following reasons: First, not all reference databases enable this option, and using it would drastically deviate the number of records obtained of some database in comparison to others. Second, in the case of Scopus database, for instance, the number of initial results would become intractable (around 27,000 records). In each reference database, the complete string was queried in the title, abstract, and keywords fields.

#### Inclusion and exclusion criteria

The selection criteria of published studies comprised the following inclusion and exclusion criteria:

- Inclusion criteria:
  I1. Papers focusing on social cognition deficits and their treatment.
  I2. Papers published between January 1, 2006, and December 31, 2016.
  I3. Papers focusing on social cognition training and remediation interventions.
- Exclusion criteria:
  E1. Grey literature, because of their unclear peer review process: editorials, extended abstracts, tutorials, tool demos, doctoral symposium papers, research abstracts, book chapters, proceedings, keynote talks, workshop reports, and technical reports.
  E2. Systematic reviews (including meta-analyses) and survey papers were not considered eligible for inclusion. We were only interested in first-hand experimental research work.
  E3. Papers from venues other than medicine, psychology, neuroscience and/or biomedicine (e.g. agriculture, environmental sciences, business, veterinary, physics, earth sciences, economy, and energy).
  E4. Papers not related to research with human subjects.
  E5. Papers based on pharmacological interventions for social cognition deficits.

Inclusion criterion I2 deserves an explanation. It is usual to consider publications published within the last ten in similar systematic mapping studies. Therefore, we started our study with publications from 2006, which provided around 150 papers as the starting point of the search. Moreover, there are two reasons to end the study in 2016. First, systematic mappings study complete years, and it is not feasible to close a year before the third quarter of the following one, mainly due to delays inherent to data management in search engines. Thus, work on the study was started during the final months of 2017. Second, performing this systematic mapping study was a vast task that continued into 2018.

In relation to exclusion criterion E1, let us highlight that it does not limit the potential to determine interest in the topic, as we have verified the same tendency in the growth of the number of publications including and excluding grey literature. For the under study, grey literature represents around 22% of annual publications.

#### Search process

**Fig 1:**
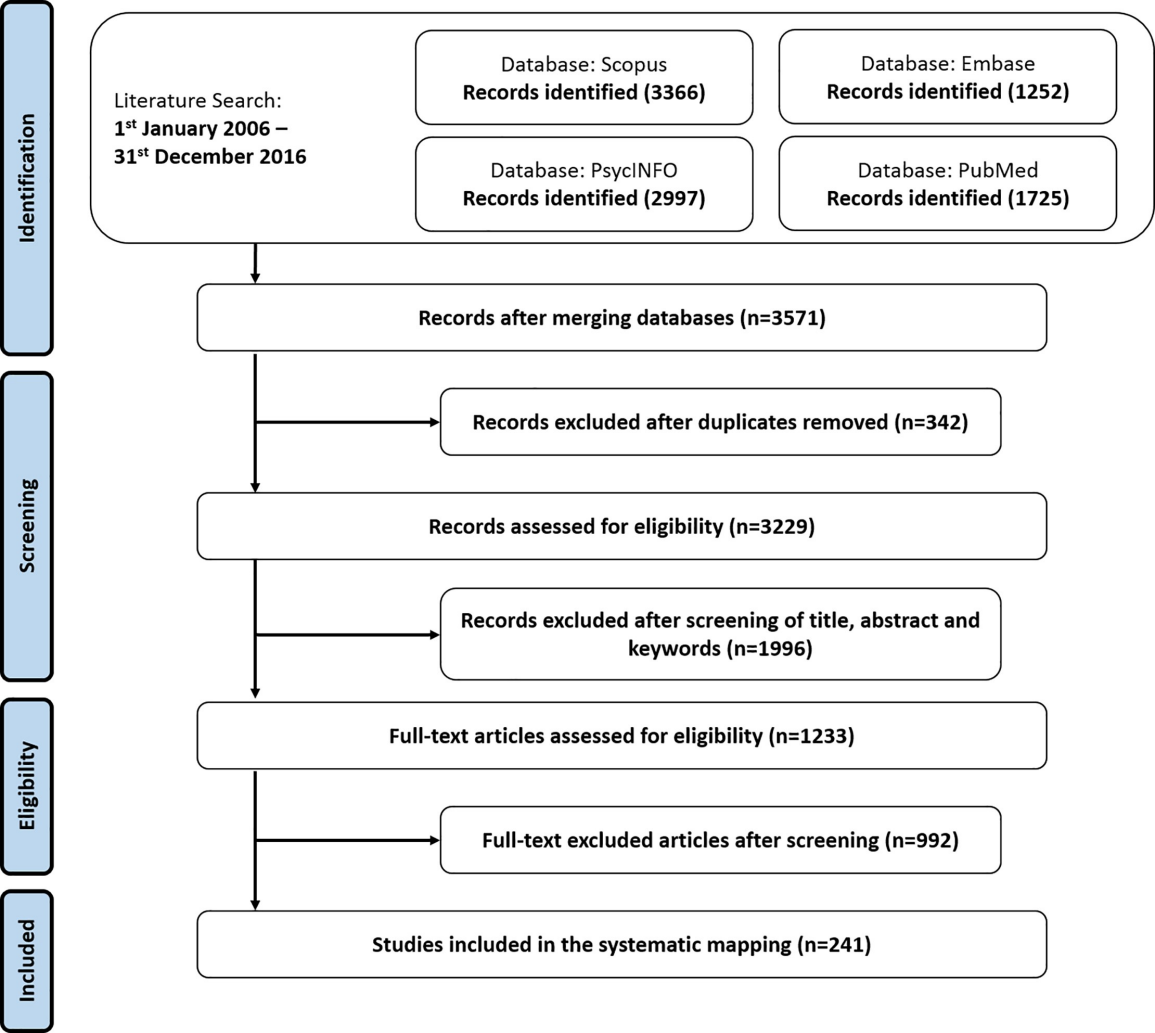
Steps for the process of extracting and selecting articles.

The steps described in Fig 1 were followed for the process of extracting and selecting articles. The search string was used on each of the four reference databases. After merging the references from each database and implementing the filters on the papers (according to inclusion and exclusion criteria), a total of 3571 papers were obtained. After eliminating repeated articles (n = 342), two researchers filtered the remaining 3229 papers independently through the screening of title, abstract and keywords. If the researcher was unsure whether to include a paper, the introduction and conclusion sections of the full content paper were read. If, despite this, the researcher was still not sure, or there was no agreement between the two researchers, the paper was presented to the other researchers and the clinical coordinator of the study for discussion and a consensus decision. This step resulted in 1233 references.

At this point, the 1233 references were filtered again by the same two researchers, who independently screened the full content of each paper. As in the preceding step, when a researcher was unsure whether to keep or remove a paper, or there was no agreement between the two researchers, the paper was presented in another session for discussion and a final decision. This step thus produced the final set of 241 references. To ensure that no references were missed during the process, a series of earlier [62–63] and more recent review papers [30–31,64], including a highly recent systematic literature review [65] were carefully studied. A full-text screening of all these papers revealed no undiscovered publications.

## Results

A final number of 241 papers (see on-line material, S1 File for a complete list of selected papers) were extracted following the previously described process. Here, the four previously presented research questions will be answered with reference to the selected literature.

RQ1. *How many papers have been published on social cognition remediation interventions*? *Is there any temporal trend*? *What is their level of scientific evidence*?

**Fig 2:**
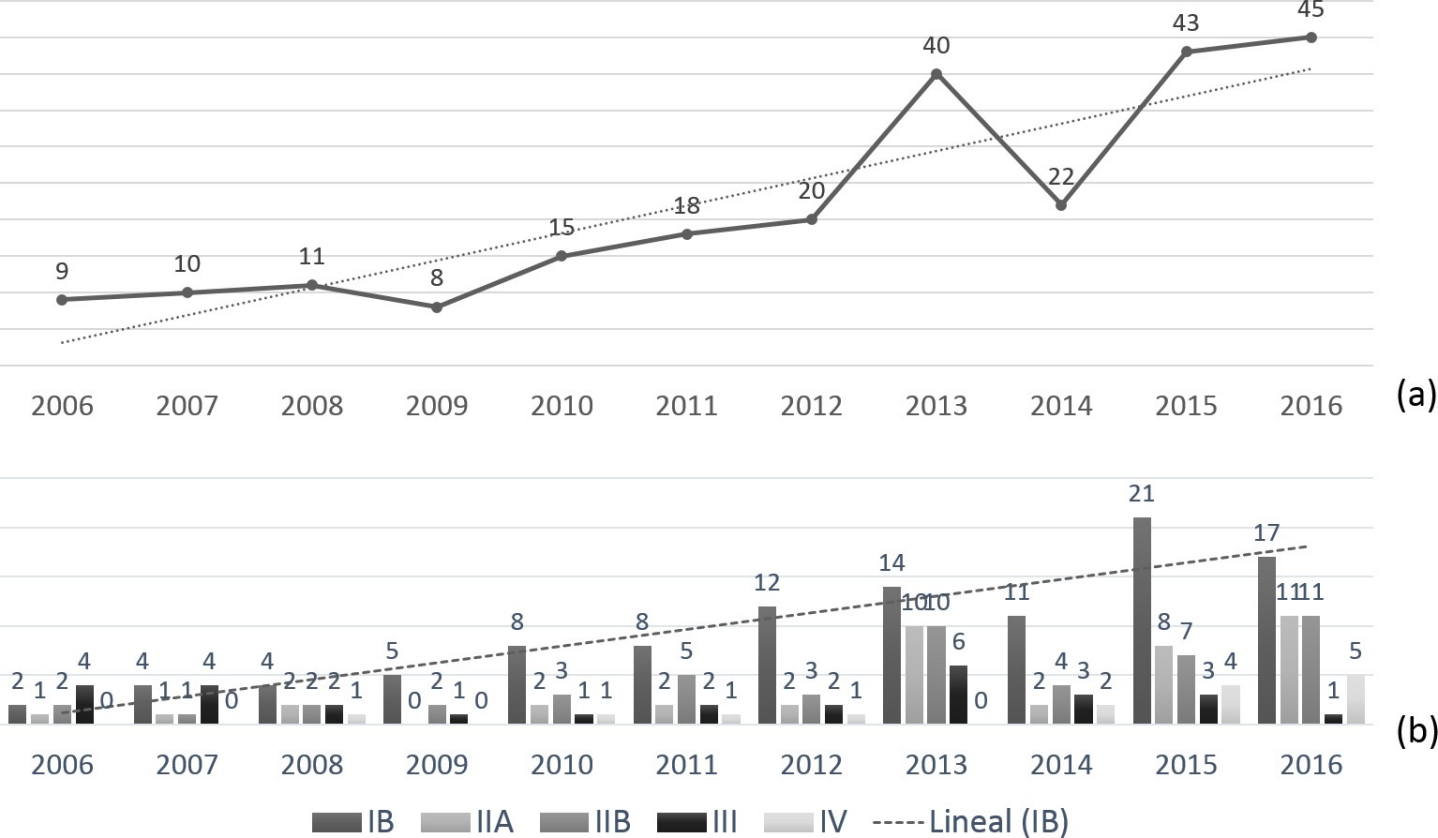
(a) Number of selected papers over publication years. (b) Number of references per year and level of scientific evidence.

As shown in Fig 2A, over the period of study, an increase was observed in the number of published papers on social cognition remediation and training programs. There was an overall progressive increase from 2006, growing from 9 papers published in 2006, to 45 papers in 2016. The greatest increase in the number of published papers was found over the last four years.

An overview of selected papers in terms of their level of scientific evidence provided data regarding the quality of published studies. As defined by the *US Agency for Healthcare Research and Quality* [66], 106 references presented IB level of evidence (evidence obtained from at least one randomized clinical trial), and 41 papers presented IIA level (obtained from at least one well-designed, non-randomized controlled prospective study). Among the remaining references, 50 studies were classified as IIB (scientific evidence obtained from at least one well-designed, quasi-experimental study), while 29 papers presented III level of evidence (well-designed observational studies). The remaining 15 papers were classified as IV (documents or opinions of expert committees and/or clinical experiences of key opinion leaders). An increase in the number of published papers on social cognition remediation and training programs with IB level of evidence was seen in the period studied, as can be seen in the IB trend line (see Fig 2B).

Regarding publication venues, *Schizophrenia Research* journal was the most popular venue, with 19 papers, followed by *Journal of Autism and Developmental Disorders* with 10 papers, *Psychiatry Research* with 8 published papers, *Behavioural and Cognitive Psychotherapy* with 7 papers, *PLoS ONE* with 6 papers, *Schizophrenia Bulletin* with 5 papers, and *BMC Psychiatry* and *Developmental Psychology* with 4 papers each. Each of the remaining journals had 3 or fewer published papers (see on-line material, S2 Table for a complete list).

RQ2. *In what areas of medical knowledge and related disciplines has research on social cognition remediation programs been conducted*?

According to the area of knowledge of the publishing journal, 132 of the 241 included papers were published in journals classified as related to medicine. As shown in Table 1, papers on medicine were mostly in the field of psychiatry, according to the *EUMS* (*European Union of Medical Specialists*) (89 papers of a total of 132 in medical specialties). Other areas of knowledge were child and adolescent psychiatry and psychotherapy (22 papers), neurology (6 papers), physical medicine and rehabilitation (6 papers), and public health medicine (4 papers).

**Table 1:** References by EUMS medical specialties.

EUMS Medical Specialty	Ref.
Psychiatry	P5, P6, P10, P16, P23, P24, P26, P30, P31, P32, P34, P36, P39, P40, P43, P44, P45, P46, P47, P48, P59, P60, P61, P62, P63, P64, P65, P67, P70, P71, P73, P79, P80, P83, P84, P86, P93, P94, P100, P101, P102, P103, P104, P109, P110, P112, P113, P117, P121, P122, P130, P131, P132, P133, P136, P140, P143, P144, P146, P148, P149, P153, P156, P158, P159, P161, P164, P172, P179, P181, P185, P186, P189, P191, P192, P202, P204, P206, P209, P212, P215, P225, P226, P230, P231, P232, P237, P238, P240
Child and Adolescent Psychiatry and Psychotherapy	P7, P8, P11, P12, P13, P18, P19, P52, P77, P81, P107, P116, P123, P124, P125, P155, P194, P201, P208, P216, P219, P235
Neurology	P2, P76, P145, P157, P165, P171
Physical Medicine and Rehabilitation	P1, P50, P174, P183, P199, P218
Public Health Medicine	P25, P126, P227, P241
Geriatrics	P120
Laboratory Medicine / Medical Biopathology	P198
Pediatrics	P90
Non-Specific	P51, P99

Furthermore, as can be seen in Table 2, according to the area of knowledge of the publishing journal, the second most frequent studied knowledge area after medicine was psychology, which included 93 papers. The remaining 16 papers were included in science, neuroscience, and education journals.

**Table 2:** References by journal knowledge area.

Knowledge Area	Ref.
Medicine	P1, P2, P5, P6, P7, P8, P10, P11, P12, P13, P16, P18, P19, P23, P24, P25, P26, P30, P31, P32, P34, P36, P39, P40, P43, P44, P45, P46, P47, P48, P50, P51, P52, P59, P60, P61, P62, P63, P64, P65, P67, P70, P71, P73, P76, P77, P79, P80, P81, P83, P84, P86, P90, P93, P94, P99, P100, P101, P102, P103, P104, P107, P109, P110, P112, P113, P116, P117, P120, P121, P122, P123, P124, P125, P126, P130, P131, P132, P133, P136, P140, P143, P144, P145, P146, P148, P149, P153, P155, P156, P157, P158, P159, P161, P164, P165, P171, P172, P174, P179, P181, P183, P185, P186, P189, P191, P192, P194, P198, P199, P201, P202, P204, P206, P208, P209, P212, P215, P216, P218, P219, P225, P226, P227, P230, P231, P232, P235, P237, P238, P240, P241
Psychology	P3, P4, P9, P14, P15, P17, P20, P21, P22, P28, P29, P33, P35, P37, P38, P41, P42, P49, P54, P55, P56, P57, P58, P69, P72, P74, P75, P78, P82, P85, P87, P88, P89, P91, P92, P95, P96, P97, P98, P105, P106, P108, P114, P118, P119, P127, P134, P135, P137, P138, P141, P147, P150, P151, P152, P154, P160, P162, P166, P167, P168, P169, P170, P173, P175, P176, P177, P178, P180, P182, P184, P187, P188, P190, P195, P196, P200, P205, P210, P211, P213, P214, P217, P220, P221, P222, P223, P224, P228, P229, P233, P234, P239
Science	P66, P68, P111, P129, P142, P163, P193, P197, P203, P207, P236
Neuroscience	P27, P53, P128, P139
Education	P115

RQ3. *Which domains of social cognition are the focus of research in social cognition remediation*?

**Fig 3:**
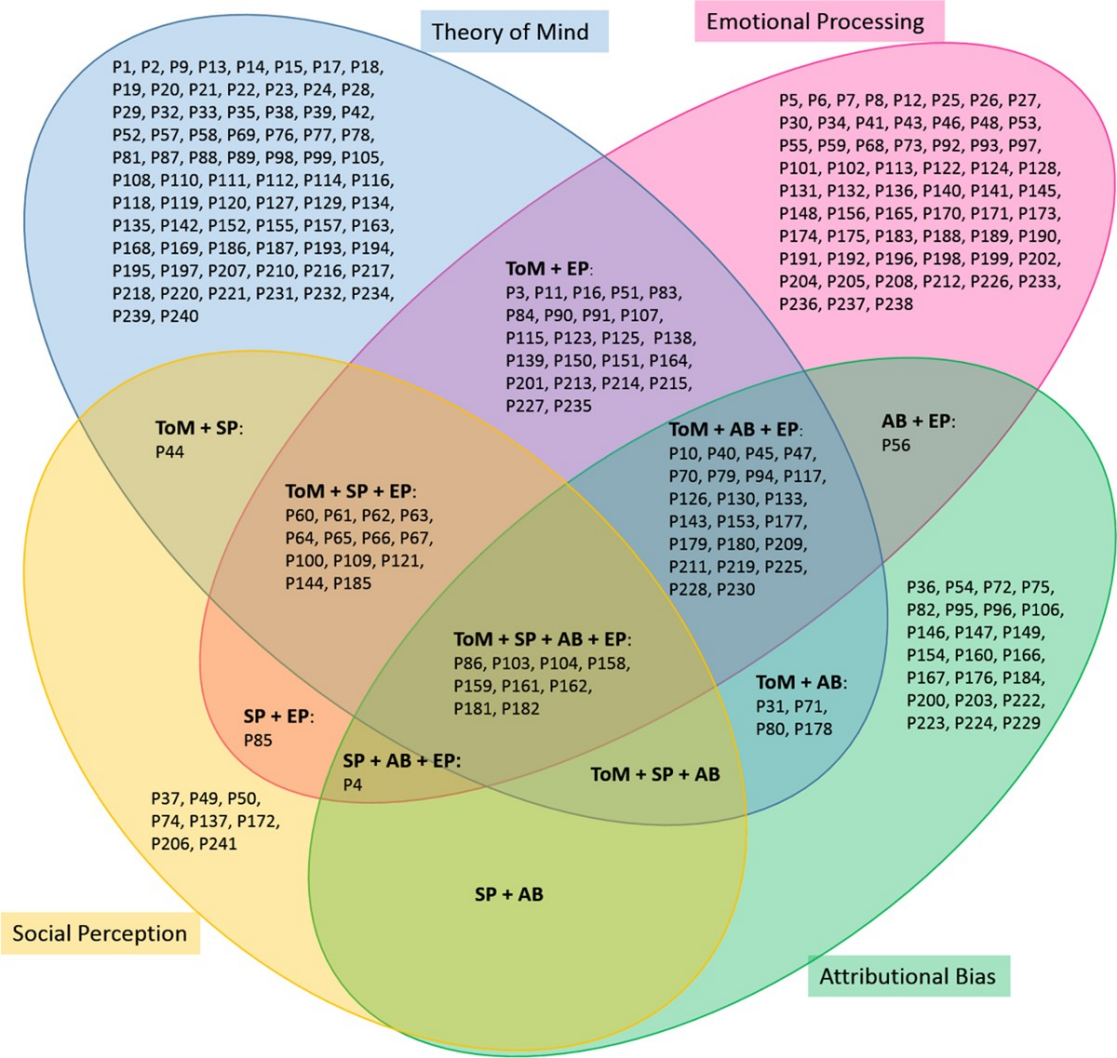
References by domains of social cognition. Venn diagram with four sets.

Theory of mind was the most frequently studied domain with 146 publications, followed by 131 articles in emotional processing, 60 articles in attributional bias and 33 papers in social perception (see Fig 3). Although most studies focus on a single domain of social cognition, some evaluate two or more domains of social cognition, as shown in Fig 3. In this case, the most frequent combination was the pair formed by theory of mind and emotional processing (23 papers), as well as the triad of theory of mind, attributional bias and emotional processing (22 papers).

**Fig 4:**
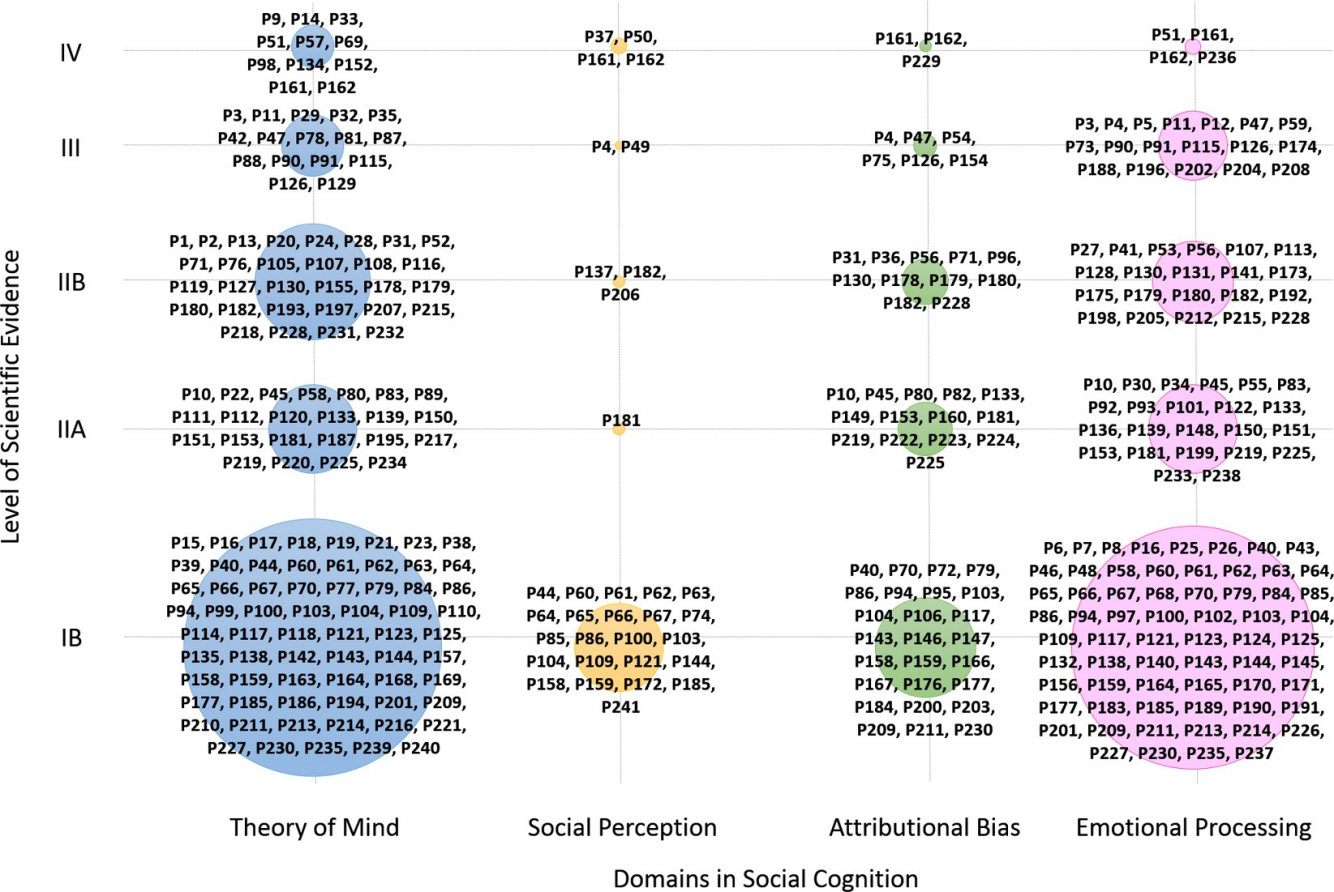
Categorization of papers in terms of social cognition domain and level of scientific evidence.

Studies with IB level of evidence were the most frequently found for each domain of social cognition. As can be observed in Fig 4, the greatest number focused on theory of mind and on emotional processing with IB level of evidence.

As a result of classifying publications according to domains of social cognition and the mental disorder they focus on, most publications in all four domains alluded to schizophrenia spectrum and other psychotic disorders. Autism spectrum disorder was the second most studied disorder (see Table 3).

**Table 3:** Mental disorders for each domain in social cognition.

Social Cognition Domain	
Mental Disorder	Ref.
**Emotion Processing**	
Schizophrenia Spectrum and Other Psychotic Disorders	P4, P10, P16, P30, P34, P45, P46, P47, P48, P59, P60, P61, P62, P63, P64, P65, P66, P67, P70, P79, P83, P84, P85, P86, P92, P94, P100, P101, P102, P103, P104, P109, P121, P122, P128, P130, P131, P132, P133, P136, P138, P140, P143, P144, P148, P153, P158, P159, P161, P162, P164, P165, P173, P177, P179, P180, P181, P182, P185, P188, P189, P191, P192, P202, P204, P209, P225, P226, P227, P228, P230, P237, P238
Autism Spectrum Disorder	P5, P7, P8, P11, P12, P26, P27, P41, P56, P93, P107, P115, P122, P123, P124, P125, P126, P175, P183, P190, P198, P201, P213, P214, P215, P219, P233, P235
Depressive Disorders	P6, P73
Feeding and Eating Disorders	P141, P212
Anxiety Disorders	P113
Attention Deficit / Hyperactivity Disorder	P43
Bipolar and Related Disorders, and Schizoaffective Disorder	P117
Disruptive, Impulse-Control, and Conduct Disorders	P51
Personality Disorders	P40
**Theory of Mind**	
Schizophrenia Spectrum and Other Psychotic Disorders	P9, P10, P14, P15, P16, P17, P28, P33, P39, P44, P45, P47, P60, P61, P62, P63, P64, P65, P66, P67, P70, P71, P79, P80, P83, P84, P86, P94, P98, P100, P103, P104, P108, P109, P121, P130, P133, P138, P142, P143, P144, P152, P153, P158, P159, P161, P162, P164, P177, P178, P179, P180, P181, P182, P185, P209, P220, P225, P227, P228, P230
Autism Spectrum Disorder	P2, P11, P13, P18, P19, P24, P42, P52, P69, P77, P81, P88, P99, P107, P110, P114, P115, P116, P123, P125, P126, P134, P135, P155, P168, P193, P194, P201, P213, P214, P215, P216, P219, P235, P239
Borderline Personality Disorder	P32, P232
Personality Disorders	P40, P57
Attention Deficit / Hyperactivity Disorder	P23
Bipolar and Related Disorders, and Schizoaffective Disorder	P117
Communication Disorders	P197
Delusional Disorder	P31
Disruptive, Impulse-Control, and Conduct Disorders	P51
Intellectual Disabilities	P1
Trauma- and Stressor-Related Disorders	P112
**Attributional Bias**	
Schizophrenia Spectrum and Other Psychotic Disorders	P4, P10, P45, P47, P70, P71, P79, P80, P86, P94, P103, P104, P130, P133, P143, P153, P158, P159, P161, P162, P177, P178, P179, P180, P181, P182, P209, P225, P228, P230
Anxiety Disorders	P72, P147, P203, P223, P224
Depressive Disorders	P106, P149, P160, P200, P229
Autism Spectrum Disorder	P56, P126, P219
Bipolar and Related Disorders, and Schizoaffective Disorder	P117
Delusional Disorder	P31
Feeding and Eating Disorders	P36
Personality Disorders	P40
**Social Perception**	
Schizophrenia Spectrum and Other Psychotic Disorders	P4, P44, P60, P61, P62, P63, P64, P65, P66, P67, P74, P85, P86, P100, P103, P104, P109, P121, P137, P144, P158, P159, P161, P162, P172, P181, P182, P185, P206, P241
Autism Spectrum Disorder	P50
Intellectual Disabilities	P49

RQ4. *What types of interventions are employed in social cognitive remediation*?

As previously stated, on the basis of previous reviews and meta-analyses, three types of programs can be distinguished [31]: targeted interventions, broad-based interventions, and non-specific interventions. The latter may influence social cognition, but do not address it directly. According to this classification, targeted interventions were the focus of the largest number of publications with 156 papers, followed by non-specific therapies with 59 publications, and broad-based interventions with 26 publications (see on-line material, S3 Table, for a complete list).

Among targeted interventions, *Social Cognition and Interaction Training* (SCIT) was the focus of 16 publications, followed by *Training of Affect Recognition* (TAR) and *Conversation-Based Intervention*, both with 7 papers, Mind *Reading* and *SummerMAX* with 6 publications each, and *Mentalization-Based Therapy* (MBT), *Micro-Expression Training Tool* (METT) and *The Theory of Mind Training* with 4 publications each (see Table 4). The complete list of targeted interventions can be found as on-line material (S3 Table).

**Table 4:** Targeted interventions in social cognitive remediation.

Targeted Interventions	Refs
Social Cognition and Interaction Training (SCIT) [67]	P10, P40, P45, P47, P94, P117, P126 (SCIT-A^a^), P153, P177, P179, P180, P209 (F-SCIT^b^), P211 (IFW-SCIT ^c^), P219 (SCIT-A^a^), P228, P230
Training of Affect Recognition (TAR) [68]	P59, P92, P128, P191, P204, P237, P238
Conversation-Based Intervention [69]	P21, P22, P38, P118, P119, P120, P186
Mind Reading [70]	P88, P115, P116, P215, P216, P233
SummerMAX [71]	P123, P124, P125, P183, P213, P214
Mentalization-Based Therapy (MBT) [72]	P32, P221, P231, P232
Micro-Expression Training Tool (METT) [73]	P131, P132, P188, P189
The Theory of Mind Training [74]	P18, P19, P81, P99

^a^ SCIT-A: Social Cognition and Interaction Training Modified for High Functioning Autism

^b^ F-SCIT: Social Cognition and Interaction Training Modified for Family-Assisted

^c^ IFW-SCIT: Social Cognition and Interaction Training Modified for Inpatient Forensic Wards

In the group of broad-based interventions, the most frequently studied therapy was *Cognitive Enhancement Therapy* (CET) with 11 publications, as shown in Table 5. The second most studied therapy was *Integrated Psychological Therapy* (IPT) with 4 articles. *Auditory Training with Cognitive Social Training plus Social Cognition Training* (AT + SCT) followed with 3 papers, and *Cognitive Pragmatic Treatment* (CPT) and *REHACOP* with 2 papers each. The rest of the papers on broad-based interventions are available in the supplementary material online (S3 Table).

**Table 5:** Broad-based interventions in social cognitive remediation.

Broad-Based Interventions	Refs
Cognitive Enhancement Therapy (CET) [75]	P60, P61, P62, P63, P64, P65, P66, P67, P100, P109, P121
Integrated Psychological Therapy (IPT) [76]	P74, P172, P206, P241
Auditory-Based Cognitive Training plus Social-Cognition Training (AT+ SCT) [77]	P101, P102, P192
Cognitive Pragmatic Treatment (CPT) [78]	P28, P76
REHACOP (*Programa de REHAbilitación COgnitiva en Psicosis*) [79]	P157, P158

Finally, in the group of non-specific interventions on social cognition (see S3 Table), the largest number of publications referred to *Cognitive and Behavioral Therapies* with 28 papers, followed by *Neurocognition Training* with 5 publications, and *Mindfulness* and *Art Therapy* with 4 papers each. The rest of the papers on non-specific interventions are available in the supplementary material online (see S3 Table).

Among the studies analyzed, 61 were based on computerized training versus 180 that used no new technologies, but rather interpersonal modality (see Table 6). It could be interesting to carry out studies comparing the effectiveness of these two types of intervention as future work.

**Table 6:** Computerized vs. in-person interventions.

**Computerized interventions**	
Targeted Interventions	P5, P26, P27, P41, P46, P48, P53, P56, P59, P88, P92, P97, P107, P115, P116, P128, P131, P132, P138, P144, P145, P160, P161, P162, P164, P165, P173, P181, P182, P185, P188, P189, P190, P191, P196, P198, P199, P202, P204, P208, P215, P216, P225, P233, P236, P237, P238
Broad-Based Interventions	P34, P70, P101, P102, P122, P192
Non-Specific Interventions	P12, P23, P39, P49, P93, P147, P149, P203
**In-person interventions**	
Targeted Interventions	P1, P2, P3, P10, P14, P16, P17, P18, P19, P20, P21, P22, P24, P25, P32, P33, P35, P36, P38, P40, P42, P43, P44, P45, P47, P51, P58, P69, P71, P72, P79, P80, P81, P82, P83, P84, P85, P86, P87, P90, P91, P94, P95, P96, P98, P99, P103, P104, P105, P108, P110, P117, P118, P119, P120, P123, P124, P125, P126, P127, P130, P133, P134, P137, P141, P150, P151, P152, P153, P154, P155, P156, P168, P169, P170, P171, P177, P178, P179, P180, P183, P186, P187, P194, P195, P197, P201, P209, P211, P212, P213, P214, P217, P218, P219, P220, P221, P222, P223, P224, P227, P228, P230, P231, P232, P234, P239, P240
Broad-Based Interventions	P15, P28, P60, P61, P62, P63, P64, P65, P66, P67, P74, P76, P100, P109, P121, P143, P157, P158, P172, P206, P241
Non-Specific Interventions	P4, P6, P7, P8, P9, P11, P13, P29, P30, P31, P37, P50, P52, P54, P55, P57, P68, P73, P75, P77, P78, P89, P106, P111, P112, P113, P114, P129, P135, P136, P139, P140, P142, P146, P148, P159, P163, P166, P167, P174, P175, P176, P184, P193, P200, P205, P207, P210, P226, P229, P235.

## Discussion

This is the first systematic mapping review carried out on remediation interventions for the enhancement of social cognition deficits. The study included papers published over a period of eleven years (2006–2016). The results show the current situation of this field of knowledge, both with the areas that have received more attention, as well as those that show little research development. The present study adds to the recently published systematic mapping review on pharmacological interventions for social cognition improvement [22], providing a global overview of the current state of knowledge in the field of social cognition remediation interventions.

A total of 241 papers were selected from the initial 3229 non-repeated publications obtained from four databases (Scopus, PsycINFO, PubMed, Embase). The number of papers published per year on social cognition training and remediation interventions clearly increased throughout the studied period, especially in the last four years. This is an indicator of the growing interest in social cognition non-pharmacological treatment approaches, which may be related to the scant effectiveness of pharmacological treatments in improving social cognition deficits.

In terms of the level of scientific evidence as defined by the *US Agency for Healthcare Research and Quality Agency* [66], of the 241 selected papers, 106 were classified as having IB level of evidence (evidence obtained from at least one randomized clinical trial) and 41 as IIA (obtained from at least one well-designed, non-randomized controlled prospective study). According to the social cognitive domain studied, studies with IB and IIA level of evidence were the most frequently found in the four domains of social cognition. IB was the highest level of evidence that could be found in our mapping review because we excluded papers with IA level of evidence (namely, meta-analyses of randomized clinical trials). Thus, publications with a high level of evidence were predominant, as an indicator of the quality of the research in the field. Moreover, given the high proportion of studies with IB level and their associated important economic costs, current interest in the subject is evident, both for researchers and different public and private funding agencies.

Referring to publication venues, it is interesting to note that most studies were disseminated in journals with a high impact factor. Journals with the highest number of papers on this topic were two journals in the first quartile of their area of knowledge: *Schizophrenia Research* with 19 papers (IF 3.986; Q1-Psychiatry), and *Journal of Autism and Developmental Disorders* with 10 papers (IF 8.321; Q1-Psychology, Developmental). Moreover, of the top eight most popular publication venues, five (comprising 42 of the 62 papers) were first-quartile journals. The fact that papers were published in high-impact journals may be related to the high level of scientific evidence previously indicated.

Regarding areas of medical knowledge and related disciplines with research on social cognition training and remediation, most publications belonged to the field of Medicine, especially to Adult Psychiatry, followed by Child and Adolescent Psychiatry and Psychotherapy. Psychology was the discipline with the second-greatest number of publications in social cognition remediation. The fact that impairments in social cognition frequently appear in numerous psychiatric disorders might explain why most studies belong to Adult and Child/Adolescent areas of Psychiatry and Psychology.

Concerning social cognition domains, most of the published papers targeted theory of mind, followed by emotional processing. Previously published reviews centered on schizophrenia and other related disorders showed that emotional processing was the focus of most publications [28–31,63]. Nevertheless, the fact that the present systematic mapping review included papers in disorders other than schizophrenia, such as autism spectrum disorders, might explain this difference. In fact, after schizophrenia and related disorders, autism spectrum disorders were the focus of most of the selected papers in this systematic mapping review. As deficits in theory of mind is a core problem in autism [80], a significant number of papers focused on this domain [64]. Much less frequent was the study of attributional bias, followed by research on social perception training. A feasible explanation for this may be related to the fact that assessment instruments for theory of mind and emotional processing may be considered more objective and less influenced by cultural aspects, as opposed to social perception assessment [81], and less influenced by some symptoms of the disorder itself, as opposed to, for example, attributional bias in patients with paranoid ideation. On the other hand, it has previously been suggested that improvements in attributional bias and social perception might be more difficult to achieve than in theory of mind and social perception [30,63]. More empirical work is needed to determine whether interventions on attributional bias and social perception can produce changes and whether these changes can be objectively measured.

The results regarding the types of social cognition training and remediation interventions show that the majority of publications refer to targeted interventions. This finding is in line with previous publications showing a greater number of papers related to targeted interventions compared to other types of interventions [31]. In the group of targeted interventions, the greatest number of papers were on *Social Cognition and Interaction Training* (SCIT). SCIT targets three domains of social cognition: theory of mind, emotional processing and attributional bias [67]. Other targeted interventions were *Training of Affect Recognition* (TAR) [68], *Conversation-Based Intervention* [69], *Mind Reading* [70], *SummerMAX* [71], *Mentalization-Based Therapy* (MBT) [72], *Micro-Expression Training Tool* (METT) [73], and *The Theory of Mind Training* [74]. Among broad-based interventions, the therapy with the largest number of papers was *Cognitive Enhancement Therapy* (CET) [75]. CET consists of computer-assisted neurocognitive and social cognitive group training to improve theory of mind, social perception and emotional processing [82]. Other less frequently published broad-based interventions were *Integrated Psychological Therapy* (IPT) [76], *Auditory Training with Cognitive Social Training plus Social Cognition Training* (AT + SCT) [77] and *REHACOP* [79]. Finally, in the group of non-specific interventions, the largest number of publications was in *Cognitive and Behavioral Therapies*. Nevertheless, although publications on certain interventions were more numerous than others, the results show the existence of a considerable number of different interventions for improving social cognition. This may be partly due to the existence of different domains of social cognition, but also due to the limited number of comparative studies.

The present systematic mapping review has some risks and limitations. One of the risks implied in all systematic mapping reviews is related to selective reporting bias [83]. To minimize this risk, four different databases were used as the source for the search process: PsycINFO, PubMed, Embase and Scopus. These provide a comprehensive list of articles encompassing the different aspects of this mapping review. Nevertheless, we should acknowledge that the decision not to truncate search terms may have led to some important articles being overlooked. It is also worth noting that it was decided to exclude grey literature (e.g. theses, internal reports, etc.) from the study. This may have impinged on the validity of the study, but it should be stressed that grey literature is generally published without a rigorous review process. Another possible risk that could have affected this mapping review is the selection bias. This is related to the criteria used to select the articles to be analyzed during the study. In order to mitigate such a risk, both the inclusion and exclusion criteria were clearly defined.

Finally, the risk of likely inaccuracy in data extraction and misclassification was mitigated as classification and extraction data processes were performed independently by two researchers, and a consensus decision including the study coordinator was made in the event of no agreement between these two researchers.

## Conclusions

To our knowledge, this is the first systematic mapping review describing social cognition remediation interventions. Our results show a growing interest in non-pharmacological treatments of social cognition, as can be demonstrated by the increase in the number of papers published over the studied period, their high level of scientific evidence, and their dissemination in journals with a high impact factor. Most studies are from the fields of psychiatry and psychology, with schizophrenia spectrum disorders and autism spectrum disorders being the two most studied conditions. Theory of mind was the most frequently studied domain of social cognition, followed by emotional processing. Targeted interventions were the most frequently studied, especially SCIT. Future research should be conducted on attributional bias and domains of social perception, with the comparative efficacy of different social cognition interventions also being an interesting future line of research.

## Supporting information

S1 Table Template for a Mapping Study Protocol. (PDF)

S2 Table Publication venues. (PDF)

S3 Table Cognitive rehabilitation programs. (PDF)

S1 File Selected papers. (PDF)
