# Supplementary material for: Social cognition remediation interventions: A systematic mapping review
Source: PLoS One. 2019 Jun 26;14(6):e0218720. doi: 10.1371/journal.pone.0218720 (PMC6594616; doi:10.1371/journal.pone.0218720)
Supplement: S1 Table — (PDF) [file pone.0218720.s001.pdf]

# S1: Template for a Mapping Study Protocol

| Section | Recommendation | Line # |
|---|---|---|
| **1. Change Record** | | |
| | This should be a list or table summarizing the main updates and changes embodied in each version of the protocol and (where appropriate), the reasons for these. | **N/A** |
| **2. Background** | | |
| | **a)** explain why there is a need for a study on this topic | **86-91** |
| | **b)** identify the topic that is to be 'scoped' in the study | **70-72** |
| | **c)** specify any research questions that will be addressed | **107-109; 117-118; 127; 150** |
| | **d)** if extending previous research on the topic, explain why a new study is needed | **N/A** |
| **3. Search Strategy** | | |
| | **a)** specify and justify basic strategy: manual search, automated search, or mixed | **172-175** |
| | **b)** for automated searches, specify search terms and compounds of these (and record results of any prototyping of the search strings) | **176-190** |
| | **c)** for automated searches, identify resources to be used (digital libraries and search engines) | **172-175** |
| | **d)** for manual searches, identify the journals and conferences to be searched | **N/A** |
| | **e)** specify the time period to be covered by the review and any reasons for your choice | **195** |
| | **f)** identify any ancillary search procedures, e.g. asking leading researchers or research groups, or accessing their web sites; or checking reference lists of primary studies | **233-240** |
| | **g)** specify how the search process is to be evaluated (e.g. against a known subset of papers; or against the results from a previous systematic review or mapping study) | **233-240** |

| Section | Recommendation | Line # |
|---|---|---|
| **4. Selection Criteria** | | |
| | **a)** identify the *inclusion* criteria for primary studies | **193-196; 208-214** |
| | **b)** identify the *exclusion* criteria | **197-207; 215-218** |
| | **c)** define how selection will be undertaken (roles of analysts) | **168-171** |
| | **d)** define how agreement among analysts will be evaluated | **221-237** |
| | **e)** define how any differences between analysts will be resolved | **221-237** |
| **5. Data Extraction** | | |
| | **a)** design data extraction form (and check via a dry run) | **176-181** |
| | **b)** specify the strategy for extracting the data and the form (paper, on-line etc.) | **176-181** |
| | **c)** identify how the data extraction process is to be undertaken and validated, particularly any data that require numerical calculations, or are subjective | **N/A** |
| **6. Synthesis** | | |
| | **a)** specify the categorization schemes to be used | **281; 287; 312; 328** |
| | **b)** assess the threats to validity (construct, internal, external), particularly constraints on the search process and deviations from standard practice | **422-433** |
| **7. Study Limitations** | | |
| | Specify residual validity issues including potential conflicts of interest (i.e. that are inherent in the context of the study, rather than arising from the plan). | **426-438** |
| **8. Reporting** | | |
| | Identify target audience, relationship to other studies, planned publications, authors of the publications. | **407-425** |
| **9. Schedule** | | |
| | Provide time estimates for all of the major steps. | **N/A** |
