# Supplementary material for: Social cognition remediation interventions: A systematic mapping review
Source: PLoS One. 2019 Jun 26;14(6):e0218720. doi: 10.1371/journal.pone.0218720 (PMC6594616; doi:10.1371/journal.pone.0218720)
Supplement: S2 Table — (PDF) [file pone.0218720.s002.pdf]

S3: Publication venues

Publication	Ref	# ref	Publication	Ref	# ref	Publication	Ref	# ref
**Schizophrenia Research**	**P45, P46, P47, P48, P59, P61, P63, P64, P86, P102, P103, P109, P121, P132, P140, P181, P189, P191, P226**	**19**	Biological Psychiatry	P73	1	Journal of Positive Behavior Interventions	P69	1
**Journal of Autism and Developmental Disorders**	**P11, P12, P18, P81, P107, P124, P125, P208, P216, P219**	**10**	BMC Research Notes	P193	1	Journal of Psychiatric and Mental Health Nursing	P232	1
**Psychiatry Research**	**P16, P40, P65, P70, P172, P179, P209, P230**	**8**	Body, Movement and Dance in Psychotherapy	P205	1	Journal of Psychiatric Research	P104	1
**Behavioural and Cognitive Psychotherapy**	**P178, P180, P211, P222, P224, P228, P229**	**7**	British Journal of Educational Psychology	P21	1	Journal of Research in Character Education	P54	1
**PLoS ONE**	**P68, P129, P142, P163, P203, P207**	**6**	Child and Family Behavior Therapy	P233	1	Journal of School Violence	P75	1
**Schizophrenia Bulletin**	**P122, P136, P143, P148, P237**	**5**	Child Development	P118	1	Journal of Special Education and Rehabilitation	P1	1
**BMC Psychiatry**	**P6, P133, P185, P212**	**4**	Chinese Mental Health Journal	P240	1	Journal of the International Neuropsychological Society	P170	1
**Developmental Psychology**	**P20, P82, P127, P234**	**4**	Clinical Case Studies	P51	1	Journal of the Society for Social Work and Research	P66	1
Autism	P13, P52, P116	3	Clinical Practice and Epidemiology in Mental Health	P227	1	Memory and Cognition	P29	1
Behaviour Research and Therapy	P72, P176, P184	3	Clinical Psychology and Psychotherapy	P152	1	Molecular Autism	P198	1
Clinical Neuropsychiatry	P32, P39, P164	3	Cognition	P195	1	Nervenheilkunde	P238	1
Clinical Schizophrenia and Related Psychoses	P83, P84, P192	3	Cognitive Behaviour Therapy	P149	1	NeuroImage: Clinical	P165	1
Frontiers in Psychiatry	P79, P112, P161	3	Cognitive Neuropsychiatry	P31	1	Neurology	P157	1
Frontiers in Psychology	P28, P38, P221	3	Community Mental Health Journal	P44	1	Neuropsychiatric Disease and Treatment	P93	1
Journal of Clinical Psychology	P9, P57, P98	3	Development and Psychopathology	P88	1	Neuropsychiatrie de l'Enfance et de l'Adolescence	P8	1
Journal of Experimental Child Psychology	P22, P119, P150	3	Early Child Development and Care	P78	1	Neuropsychiatry	P23	1
Journal of Head Trauma Rehabilitation	P76, P145, P171	3	Early Education and Development	P3	1	Neuropsychologia	P53	1
Neuropsychological Rehabilitation	P17, P108, P138	3	Early Intervention in Psychiatry	P10	1	Journal of Deaf Studies and Deaf Education	P218	1
Psychiatric Services	P60, P94, P100	3	Estudos de Psicologia	P182	1	NPJ Schizophrenia	P158	1
Psychological Medicine	P15, P67, P196	3	European Child and Adolescent Psychiatry	P7	1	Patient Education and Counseling	P25	1
Schizophrenia Research: Cognition	P144, P159, P225	3	European Journal of Developmental Psychology	P91	1	Progress in Neuro-Psychopharmacology and Biological Psychiatry	P113	1
Australasian Psychiatry	P130, P153	2	First Language	P210	1	Psychiatry Research - Neuroimaging	P101	1
British Journal of Clinical Psychology	P177, P188	2	Focus on Autism and Other Developmental Disabilities	P215	1	PsychNology Journal	P5	1
British Journal of Psychiatry	P26, P156	2	Frontiers in Human Neuroscience	P162	1	Psychological Research	P200	1
Chinese Journal of Clinical Psychology	P168, P239	2	Infancia y Aprendizaje	P187	1	Psychological Science	P58	1
Cognitive Development	P87, P217	2	Infancy	P90	1	Psychologie Française	P37	1

Computers in Human Behavior	P41, P56	2	Infant Mental Health Journal	P194	1	Psychology and Psychotherapy	P220	1
Journal of Affective Disorders	P36, P117	2	International Journal of High Risk Behaviors and Addiction	P146	1	Psychology in the Schools	P214	1
Journal of Behavior Therapy and Experimental Psychiatry	P80, P147	2	International Journal of Psychology and Psych. Therapy	P74	1	Psychosis	P206	1
Journal of Cognition and Development	P89, P105	2	International Journal of Rehabilitation Research	P174	1	Psychotherapy	P55	1
Journal of the American Academy of Child and Adolescent Psychiatry	P77, P201	2	International Journal of Telerehabilation	P236	1	Psychotherapy and Psychosomatics	P114	1
Personality and Individual Differences	P97, P166	2	Iranian Journal of Child Neurology	P2	1	Remedial and Special Education	P115	1
Psicothema	P35, P134	2	Iranian Journal of Psychiatry and Clinical Psychology	P110	1	Revista de Logopedia, Foniatría y Audiología	P197	1
Psychiatric Rehabilitation Journal	P30, P71	2	Journal de Therapie Comportementale et Cognitive	P14	1	Revista de Psicologia Social	P96	1
Rehabilitacion Psicosocial	P4, P173	2	Journal for Specialists in Group Work	P223	1	Revista de Psiquiatria Clinica	P202	1
Research in Autism Spectrum Disorders	P123, P155	2	Journal of Abnormal Psychology	P160	1	Science	P111	1
Social Development	P151, P169	2	Journal of Attention Disorders	P43	1	Shanghai Archives of Psychiatry	P34	1
Social Neuroscience	P92, P128	2	Journal of Child Psychology and Psychiatry and All. Disc.	P235	1	Social Cognitive and Affective Neuroscience	P135	1
Aging and Mental Health	P186	1	Journal of Clinical & Adolescent Psychology	P213	1	Spanish Journal of Psychology	P85	1
American Journal of Psychiatric Rehabilitation	P131	1	Journal of Consulting and Clinical Psychology	P106	1	Sport Psychologist	P154	1
Applied Neuropsychology	P137	1	Journal of Contemporary Psychotherapy	P33	1	Therapeutic Recreation Journal	P49	1
Archives of General Psychiatry	P62	1	Journal of Developmental and Physical Disabilities	P183	1	Topics in Language Disorders	P50	1
Archives of Gerontology and Geriatrics	P120	1	Journal of Experimental Psychology: General	P42	1	Trials	P99	1
Art Therapy	P175	1	Journal of Family Psychology	P167	1	Work	P126	1
Autism Research	P19	1	Journal of Forensic Psychiatry and Psychology	P231	1	World Health	P241	1
Behavior Therapy	P95	1	Journal of Health Psychology	P141	1	World Journal of Biological Psychiatry	P204	1
Behavioral and Brain Functions	P139	1	Journal of Nonverbal Behavior	P190	1	Zeitschrift fur Psychiatrie, Psychologie und Psychotherapie	P24	1
Behavioral Neuroscience	P27	1	Journal of Intellectual Disability Research	P199	1			
