# Supplementary material for: Social cognition remediation interventions: A systematic mapping review
Source: PLoS One. 2019 Jun 26;14(6):e0218720. doi: 10.1371/journal.pone.0218720 (PMC6594616; doi:10.1371/journal.pone.0218720)
Supplement: S3 Table — (PDF) [file pone.0218720.s003.pdf]

S4: Cognitive Rehabilitation Programs

Targeted Interventions

Named Intervention Program	Ref	#ref
Social Cognition and Interaction Training (SCIT)	P10, P40, P45, P47, P94, P117, P153, P177, P179, P180, P228, P230	12
Training of Affect Recognition (TAR)	P59, P92, P128, P191, P204, P237, P238	7
Conversation-Based Intervention	P21, P22, P38, P118, P119, P120, P186	7
Mind Reading	P88, P115, P116, P215, P216, P233	6
summerMAX	P123, P124, P125, P183, P213, P214	6
Mentalization-Based Therapy (MBT)	P32, P221, P231, P232	4
Micro-Expression Training Tool (METT)	P131, P132, P188, P189	4
The Theory of Mind Training	P18, P19, P81, P99	4
Metacognitive Reflective and Insight Therapy (MERIT)	P33, P96, P98	3
Metacognitive Training (MCT)	P80, P170, P220	3
Social Cognitive Skills Training (SCST)	P86, P103, P104	3
Social Cognitive Training Program (PECS)	P83, P84, P85	3
Attentional Shaping Program	P46, P48	2
Cognitive Remediation and Emotion Skills Training (CREST)	P141, P212	2
Cognitive Remediation of Theory of Mind (ToMRemed)	P14, P108	2
Emotion and ToM Imitation Training (ETIT)	P138, P164	2
Frankfurt Test and Training of Facial Affect Recognition (FEFA)	P26, P27	2
Mental-State Reasoning Training for Social Cognitive Impairment (SoCog-MSRT)	P130, P133	2
Metacognitive and Social Cognitive Skills Program (MSCT)	P181, P182	2
Online Social Cognitive Program "SocialVille"	P144, P185	2
RC2S (Remédiation Cognitive de la Cognition Sociale) Program	P161, P162	2
Social Cognition and Interaction Training Modified for High Functioning Autism (SCIT-A)	P126, P219	2
Theory of Mind Intervention (ToMI)	P15, P17	2
Augmented Reality-based Video-Modeling with Storybook (ARVMS)	P41	1
Attributional Style Training "Positive Interpretation Training"	P95	1
Coaching and Rewarding Emotional Skills (CARES)	P51	1
Cognitive-Emotional Rehabilitation (REC)	P227	1
Emotional Management Training (EMT)	P43	1
Emotion Recognition Training (SoCog-ERT)	P133	1
E-Motional Training	P225	1
FaceGen	P202	1
Gaïa s-face program	P79	1
JeStiMulE	P198	1
Let's Talk about Emotions!	P151	1
LIFEisGAME	P5	1
Mary/Eddie/Bill (MEB)	P178	1
Metacognition-Oriented Social Skills Training (MOSST)	P152	1
Microexpression Recognition Training Tool (The MIX Program)	P190	1
Nonverbal communication, Emotion recognition, and Theory of mind Training (Seaver-NETT)	P201	1
Social Cognition and Interaction Training Modified for Family-Assisted (F-SCIT)	P209	1
Social Cognition and Interaction Training Modified for Inpatient Forensic Wards (IFW-SCIT)	P211	1
Social Cognition Enhancement Training (SCET)	P44	1
Theory-of-Mind based Social Skills Group Training for Children and Adolescents with ASD (TOMTASS)	P24	1
Understanding Social Situations (USS)	P71	1

(Unnamed) Intervention Focused on	Ref	#ref
Theory of Mind	P1, P2, P3, P15, P16, P20, P35, P42, P58, P69, P87, P90, P91, P105, P107, P110, P127, P134, P150, P155, P168, P169, P187, P194, P195, P197, P217, P218, P234, P239, P240	31
Attributional Bias	P36, P56, P72, P82, P154, P160, P203, P222, P223, P224	10
Emotional Processing	P15, P16, P25, P53, P56, P90, P91, P97, P107, P145, P150, P156, P165, P171, P173, P196, P199, P208, P236	19
Social Perception	P137, P107	2

Broad-Based Intervention	Ref	#ref
Cognitive Enhancement Therapy (CET)	P60, P61, P62, P63, P64, P65, P66, P67, P100, P109, P121	11
Integrated Psychological Therapy (IPT)	P74, P172, P206, P241	4
Auditory-Based Cognitive Training plus Social-Cognition Training (AT+ SCT)	P101, P102, P192	3
Cognitive Pragmatic Treatment (CPT)	P28, P76	2
REHACOP (Programa de REHAbilitación COgnitiva en Psicosis)	P157, P158	2
Computerized Drill Training (CDT) Program	P34	1
Mind Reading: Interactive Guide to Emotions (MRIGE)	P122	1
Integrated Neurocognitive Therapy (INT)	P143	1
Neuropersonal Trainer- Mental Health (NPT + MH)	P70	1

Non-Specific Treatment of Social Cognition	Ref	#ref
Cognitive and Behavioral Therapies	P6, P7, P8, P9, P11, P12, P13, P31, P50, P54, P57, P73, P75, P89, P93, P106, P113, P136, P146, P147, P149, P166, P176, P184, P200, P210, P226, P229	28
Neurocognition Training	P23, P30, P39, P140, P148	5
Art Therapy	P114, P142, P175, P205	4
Mindfulness	P52, P112, P139, P207	4
Physical activity	P37, P77, P148	3
Reading	P29, P111, P163	3
Films	P159, P235	2
Meditation	P129, P135	2
Other psychotherapies	P55, P78	2
Psychoeducation	P167, P174	2
Psychosocial and Employment Rehabilitation	P4, P49	2
Animal-Assisted Therapy	P193	1
Language Therapy	P68	1
