# Supplementary material for: Social cognition remediation interventions: A systematic mapping review
Source: PLoS One. 2019 Jun 26;14(6):e0218720. doi: 10.1371/journal.pone.0218720 (PMC6594616; doi:10.1371/journal.pone.0218720)
Supplement: S1 File — (PDF) [file pone.0218720.s004.pdf]

S2: Selected papers

P1 Adibsereshki, N., Abdolahzadeh, M., Karmilo, M., & Hasanzadeh, M. (2014). The effectiveness of theory of mind training on the adaptive behavior of students with intellectual disability. *Journal of Special Education and Rehabilitation*, *15*(1–2). https://doi.org/10.2478/jser-2014-0006

P2 Adibsereshki, N., Nesayan, A., Asadi Gandomani, R., & Karimlou, M. (2015). The effectiveness of theory of mind training on the social skills of children with high functioning autism spectrum disorders. *Iranian Journal of Child Neurology*, *9*(3), 40–49.

P3 Allen, J. R., & Kinsey, K. (2013). Teaching theory of mind. *Early Education & Development*, *24*(6), 865–876. https://doi.org/10.1080/10409289.2013.745182

P4 Álvarez, J. C., Tourinõ, R., Abelleira, C., Fernańdez, J., Baena, E., Giráldez, A., & Bordón, R. (2013). Social cognition and schizophrenia: Differences between users of psychosocial rehabilitation day center and from a supported employment program. *Rehabilitacion Psicosocial*, *10*(2), 4–9.

P5 Alves, S., Marques, A., Queirós, C., & Orvalho, V. (2013). LIFEisGAME prototype: A serious game about emotions for children with autism spectrum disorders. *PsychNology Journal*, *11*(3), 191–211.

P6 Babl, A., grosse Holtforth, M., Heer, S., Lin, M., Stähli, A., Holstein, D., … Caspar, F. (2016). Psychotherapy integration under scrutiny: investigating the impact of integrating emotion-focused components into a CBT-based approach: a study protocol of a randomized controlled trial. *BMC Psychiatry*, *16*(1), 423. https://doi.org/10.1186/s12888-016-1136-7

P7 Baghdadli, A., Brisot-Dubois, J., Picot, M.-C., & Michelon, C. (2010). Comparaison de l’effet de deux interventions prosociales sur l’évolution des capacités d’identification des expressions faciales et du raisonnement social d’enfants avec un syndrome d’Asperger ou autisme de haut niveau. *Neuropsychiatrie de l’Enfance et de l’Adolescence*, *58*(8), 456–462. https://doi.org/10.1016/j.neurenf.2010.06.009

P8 Baghdadli, A., Brisot, J., Henry, V., Michelon, C., Soussana, M., Rattaz, C., & Picot, M. C. (2013). Social skills improvement in children with high-functioning autism: a pilot randomized controlled trial. *European Child & Adolescent Psychiatry*, *22*(7), 433–442. https://doi.org/10.1007/s00787-013-0388-8

P9 Bargenquast, R., Schweitzer, R. D., & Drake, S. (2015). Reawakening reflective capacity in the psychotherapy of schizophrenia: A case study. *Journal of Clinical Psychology*, *71*(2), 136–145. https://doi.org/10.1002/jclp.22149

P10 Bartholomeusz, C. F., Allott, K., Killackey, E., Liu, P., Wood, S. J., & Thompson, A. (2013). Social cognition training as an intervention for improving functional outcome in first-episode psychosis: A feasibility study. *Early Intervention in Psychiatry*, *7*(4), 421–426. https://doi.org/10.1111/eip.12036

P11 Bauminger-Zviely, N., Eden, S., Zancanaro, M., Weiss, P. L., & Gal, E. (2013). Increasing social engagement in children with high-functioning autism spectrum disorder using collaborative technologies in the school environment. *Autism*, *17*(3), 317–339. https://doi.org/10.1177/1362361312472989

P12 Bauminger, N. (2007a). Brief report: Group social-multimodal intervention for HFASD. *Journal of Autism and Developmental Disorders*, *37*(8), 1605–1615. https://doi.org/10.1007/s10803-006-0246-3

P13 Bauminger, N. (2007b). Brief report: Individual social-multi-modal intervention for HFASD. *Journal of Autism and Developmental Disorders*, *37*(8), 1593–1604. https://doi.org/10.1007/s10803-006-0245-4

P14 Bazin, N., Passerieux, C., & Hardy-Bayle, M.-C. (2010). ToMRemed : une technique de remédiation cognitive centrée sur la théorie de l’esprit pour les patients schizophrènes. *Journal de Thérapie Comportementale et Cognitive*, *20*(1), 16–21. https://doi.org/10.1016/j.jtcc.2010.02.001

P15 Bechi, M., Bosia, M., Spangaro, M., Buonocore, M., Cocchi, F., Pigoni, A., … Cavallaro, R. (2015). Combined social cognitive and neurocognitive rehabilitation strategies in schizophrenia: Neuropsychological and psychopathological influences on theory of mind improvement. *Psychological Medicine*, *45*(15), 3147–3157. https://doi.org/10.1017/S0033291715001129

P16 Bechi, M., Riccaboni, R., Ali, S., Fresi, F., Buonocore, M., Bosia, M., … Cavallaro, R. (2012). Theory of mind and emotion processing training for patients with schizophrenia: Preliminary findings. *Psychiatry Research*, *198*(3), 371–377. https://doi.org/10.1016/j.psychres.2012.02.004

P17 Bechi, M., Spangaro, M., Bosia, M., Zanoletti, A., Fresi, F., Buonocore, M., … Cavallaro, R. (2013). Theory of mind intervention for outpatients with schizophrenia. *Neuropsychological Rehabilitation*, *23*(3), 383–400. https://doi.org/10.1080/09602011.2012.762751

P18 Begeer, S., Gevers, C., Clifford, P., Verhoeve, M., Kat, K., Hoddenbach, E., & Boer, F. (2011). Theory of mind training in children with autism: A randomized controlled trial. *Journal of Autism and Developmental Disorders*, *41*(8), 997–1006. https://doi.org/10.1007/s10803-010-1121-9

P19 Begeer, S., Howlin, P., Hoddenbach, E., Clauser, C., Lindauer, R., Clifford, P., … Koot, H. M. (2015). Effects and moderators of a short theory of mind intervention for children with autism spectrum disorder: A randomized controlled trial. *Autism Research*, *8*(6), 738–748. https://doi.org/10.1002/aur.1489

P20 Benson, J. E., Sabbagh, M. A., Carlson, S. M., & Zelazo, P. D. (2013). Individual differences in executive functioning predict preschoolers’ improvement from theory-of-mind training. *Developmental Psychology*, *49*(9), 1615–1627. https://doi.org/10.1037/a0031056

P21 Bianco, F., & Lecce, S. (2016). Translating child development research into practice: Can teachers foster children's theory of mind in primary school? *British Journal of Educational Psychology*, *86*(4), 592–605. https://doi.org/10.1111/bjep.12125

P22 Bianco, F., Lecce, S., & Banerjee, R. (2016). Conversations about mental states and theory of mind development during middle childhood: A training study. *Journal of Experimental Child Psychology*, *149*, 41–61. https://doi.org/10.1016/j.jecp.2015.11.006

P23 Bigorra, A., Garolera, M., Guijarro, S., & Hervas, A. (2016). Impact of working memory training on hot executive functions (decision-making and theory of mind) in children with ADHD: a randomized controlled trial. *Neuropsychiatry*, *6*(5). https://doi.org/10.4172/Neuropsychiatry.1000147

P24 Biscaldi, M., Paschke-Müller, M., Rauh, R., & Schaller, U. M. (2016). Evaluation des Freiburger TOMTASS - Ein soziales Kompetenztraining mit Schwerpunkt auf Theory of Mind für Kinder und Jugendliche mit hochfunktionalen Autismus-Spektrum-Störungen. *Zeitschrift Für Psychiatrie, Psychologie Und Psychotherapie*, *64*, 269–275. https://doi.org/10.1024/1661-4747/a000288

P25 Blanch-Hartigan, D. (2012). An effective training to increase accurate recognition of patient emotion cues. *Patient Education and Counseling*, *89*(2), 274–280. https://doi.org/10.1016/j.pec.2012.08.002

P26 Bölte, S., Ciaramidaro, A., Schlitt, S., Hainz, D., Kliemann, D., Beyer, A., … Walter, H. (2015). Training-induced plasticity of the social brain in autism spectrum disorder. *The British Journal of Psychiatry*, *207*(2), 149–157. https://doi.org/10.1192/bjp.bp.113.143784

P27 Bölte, S., Hubl, D., Feineis-Matthews, S., Prvulovic, D., Dierks, T., & Poustka, F. (2006). Facial affect recognition training in autism: Can we animate the fusiform gyrus? *Behavioral Neuroscience*, *120*(1), 211–216. https://doi.org/10.1037/0735-7044.120.1.211

P28 Bosco, F. M., Gabbatore, I., Gastaldo, L., & Sacco, K. (2016). Communicative-pragmatic treatment in schizophrenia: a pilot study. *Frontiers in Psychology*, *7*. https://doi.org/10.3389/fpsyg.2016.00166

P29 Bowes, A., & Katz, A. (2015). Metaphor creates intimacy and temporarily enhances theory of mind. *Memory & Cognition*, *43*(6), 953–963. https://doi.org/10.3758/s13421-015-0508-4

P30 Bowie, C. R., Grossman, M., Gupta, M., Holshausen, K., & Best, M. W. (2016). Action-based cognitive remediation for individuals with serious mental illnesses: Effects of real-world simulations and goal setting on functional and vocational outcomes. *Psychiatric Rehabilitation Journal*. https://doi.org/10.1037/prj0000189

P31 Brakoulias, V., Langdon, R., Sloss, G., Coltheart, M., Meares, R., & Harris, A. (2008). Delusions and reasoning: A study involving cognitive behavioural therapy. *Cognitive Neuropsychiatry*, *13*(2), 148–165. https://doi.org/10.1080/13546800801900587

P32 Brüne, M., Dimaggio, G., & Edel, M.-A. (2013). Mentalization-based group therapy for inpatients with borderline personality disorder: Preliminary findings. *Clinical Neuropsychiatry*, *10*(5), 196–201.

P33 Buck, K. D., & George, S. E. (2016). Metacognitive reflective and insight therapy for a person who gained maximal levels of metacognitive capacity and was able to terminate therapy. *Journal of Contemporary Psychotherapy*, *46*(4), 187–195. https://doi.org/10.1007/s10879-016-9322-y

P34 Byrne, L. K., Pan, L., Mccabe, M., Mellor, D., & Xu, Y. (2015). Assessment of a six-week computer-based remediation program for social cognition in chronic schizophrenia. *Shanghai Archives of Psychiatry*, *27*(5), 296–306. https://doi.org/10.11919/j.issn.1002-0829.215095

P35 Carbonero Martín, M. Á., Sáiz Manzanares, M. C., & Román Sánchez, J. M. (2013). Effect of a metacognitive training program of mentalist skills. *Psicothema*, *25*(1), 31–37. https://doi.org/10.7334/psicothema2011.192

P36 Cardi, V., Esposito, M., Bird, G., Rhind, C., Yiend, J., Schifano, S., … Treasure, J. (2015). A preliminary investigation of a novel training to target cognitive biases towards negative social stimuli in anorexia nervosa. *Journal of Affective Disorders*, *188*, 188–193. https://doi.org/10.1016/j.jad.2015.08.019

P37 Carlier, M., Mainguet, B., & Delevoye-Turrell, Y. (2016). Cognitive exercise through body movement: Using a fun and short neuropsychological tool to adapt physical activity and enhance pleasure in individuals suffering from mental illnesses. *Psychologie Française*, *61*(4), 349–359. https://doi.org/10.1016/j.psfr.2015.05.004

P38 Cavallini, E., Bianco, F., Bottiroli, S., Rosi, A., Vecchi, T., & Lecce, S. (2015). Training for generalization in theory of mind: a study with older adults. *Frontiers in Psychology*, *6*. https://doi.org/10.3389/fpsyg.2015.01123

P39 Cavallo, M., Trivelli, F., Adenzato, M., Bidoia, E., Giaretto, R. M., Oliva, F., & Luca Ostacoli, Anisa Sala, R. L. P. (2013). Do neuropsychological and social cognition abilities in schizophrenia change after intensive cognitive training? A pilot study. *Clinical Neuropsychiatry*, *10*(5), 202–221.

P40 Chan, R. C. K., Gao, X., Li, X., Li, H., Cui, J., Deng, Y., & Wang, Y. (2010). The social cognition and interaction training (SCIT): An extension to individuals with schizotypal personality features. *Psychiatry Research*, *178*(1), 208–210. https://doi.org/10.1016/j.psychres.2010.03.017

P41 Chen, C.-H., Lee, I.-J., & Lin, L.-Y. (2016). Augmented reality-based video-modeling storybook of nonverbal facial cues for children with autism spectrum disorder to improve their perceptions and judgments of facial expressions and emotions. *Computers in Human Behavior*, *55*, 477–485. https://doi.org/10.1016/j.chb.2015.09.033

P42 Chevallier, C., Parish-Morris, J., Tonge, N., Le, L., Miller, J., & Schultz, R. T. (2014). Susceptibility to the audience effect explains performance gap between children with and without autism in a theory of mind task.

Journal of Experimental Psychology: General, *143*(3), 972–979. https://doi.org/10.1037/a0035483

P43 Choi, E. S., & Lee, W. K. (2015). Comparative effects of emotion management training and social skills training in Korean children with ADHD. *Journal of Attention Disorders*, *19*(2), 138–146. https://doi.org/10.1177/1087054713496460

P44 Choi, K.-H., & Kwon, J.-H. (2006). Social cognition enhancement training for schizophrenia: A preliminary randomized controlled trial. *Community Mental Health Journal*, *42*(2), 177–187. https://doi.org/10.1007/s10597-005-9023-6

P45 Combs, D. R., Adams, S. D., Penn, D. L., Roberts, D., Tiegreen, J., & Stem, P. (2007). Social cognition and interaction training (SCIT) for inpatients with schizophrenia spectrum disorders: Preliminary findings. *Schizophrenia Research*, *91*(1–3), 112–116. https://doi.org/10.1016/j.schres.2006.12.010

P46 Combs, D. R., Chapman, D., Waguspack, J., Basso, M. R., & Penn, D. L. (2011). Attention shaping as a means to improve emotion perception deficits in outpatients with schizophrenia and impaired controls. *Schizophrenia Research*, *127*(1–3), 151–156. https://doi.org/10.1016/j.schres.2010.05.011

P47 Combs, D. R., Penn, D. L., Tiegreen, J. A., Nelson, A., Ledet, S. N., Basso, M. R., & Elerson, K. (2009). Stability and generalization of social cognition and interaction training (SCIT) for schizophrenia: Six-month follow-up results. *Schizophrenia Research*, *112*(1–3), 196–197. https://doi.org/10.1016/j.schres.2009.04.010

P48 Combs, D. R., Tosheva, A., Penn, D. L., Basso, M. R., Wanner, J. L., & Laib, K. (2008). Attentional-shaping as a means to improve emotion perception deficits in schizophrenia. *Schizophrenia Research*, *105*(1–3), 68–77. https://doi.org/10.1016/j.schres.2008.05.018

P49 Cory, L., Dattilo, J., & Williams, R. (2006). Effects of a leisure education program on social knowledge and skills of youth with cognitive disabilities. *Therapeutic Recreation Journal*, *40*(3), 144–164.

P50 Crooke, P. J., Winner, M. G., & Olswang, L. B. (2016). Thinking socially. *Topics in Language Disorders*, *36*(3), 284–298. https://doi.org/10.1097/TLD.0000000000000094

P51 Datyner, A., Kimonis, E. R., Hunt, E., & Armstrong, K. (2016). Using a novel emotional skills module to enhance empathic responding for a child with conduct disorder with limited prosocial emotions. *Clinical Case Studies*, *15*(1), 35–52. https://doi.org/10.1177/1534650115588978

P52 de Bruin, E. I., Blom, R., Smit, F. M., van Steensel, F. J., & Bogels, S. M. (2015). MYmind: Mindfulness training for youngsters with autism spectrum disorders and their parents. *Autism*, *19*(8), 906–914. https://doi.org/10.1177/1362361314553279

P53 DeGutis, J., DeNicola, C., Zink, T., McGlinchey, R., & Milberg, W. (2011). Training with own-race faces can improve processing of other-race faces: Evidence from developmental prosopagnosia. *Neuropsychologia*, *49*(9), 2505–2513. https://doi.org/10.1016/j.neuropsychologia.2011.04.031

P54 Devlin, R. S., & Gibbs, J. C. (2010). Responsible adult culture (RAC): Cognitive and behavioral changes at a community-based correctional facility. *Journal of Research in Character Education*, *8*(1), 1–20.

P55 Diamond, G. M., Shahar, B., Sabo, D., & Tsvieli, N. (2016). Attachment-based family therapy and emotion-focused therapy for unresolved anger: the role of productive emotional processing. *Psychotherapy*, *53*(1), 34–44. https://doi.org/10.1037/pst0000025

P56 Didehbani, N., Allen, T., Kandalaft, M., Krawczyk, D., & Chapman, S. (2016). Virtual reality social cognition training for children with high functioning autism. *Computers in Human Behavior*, *62*, 703–711. https://doi.org/10.1016/j.chb.2016.04.033

P57 Dimaggio, G., D'Urzo, M., Pasinetti, M., Salvatore, G., Lysaker, P. H., Catania, D., & Popolo, R. (2015). Metacognitive interpersonal therapy for co-occurrent avoidant personality disorder and substance abuse. *Journal of Clinical Psychology*, *71*(2), 157–166. https://doi.org/10.1002/jclp.22151

P58 Ding, X. P., Wellman, H. M., Wang, Y., Fu, G., & Lee, K. (2015). Theory-of-mind training causes honest young children to lie. *Psychological Science*, *26*(11), 1812–1821. https://doi.org/10.1177/0956797615604628

P59 Drusch, K., Stroth, S., Kamp, D., Frommann, N., & Wölwer, W. (2014). Effects of training of affect recognition on the recognition and visual exploration of emotional faces in schizophrenia. *Schizophrenia Research*, *159*(2–3), 485–490. https://doi.org/10.1016/j.schres.2014.09.003

P60 Eack, S., Greenwald, D., Hogarty, S., Cooley, S., DiBarry, A. L., Montrose, D., & Keshavan, M. (2009). Cognitive enhancement therapy for early-course schizophrenia: Effects of a two-year randomized controlled trial. *Psychiatric Services*, *60*(11). https://doi.org/10.1176/appi.ps.60.11.1468

P61 Eack, S. M., Greenwald, D. P., Hogarty, S. S., & Keshavan, M. S. (2010). One-year durability of the effects of cognitive enhancement therapy on functional outcome in early schizophrenia. *Schizophrenia Research*, *120*(1–3), 210–216. https://doi.org/10.1016/j.schres.2010.03.042

P62 Eack, S. M., Hogarty, G. E., Cho, R. Y., Prasad, K. M. R., Greenwald, D. P., Hogarty, S. S., & Keshavan, M. S. (2010). Neuroprotective effects of cognitive enhancement therapy against gray matter loss in early schizophrenia. *Archives of General Psychiatry*, *67*(7), 674. https://doi.org/10.1001/archgenpsychiatry.2010.63

P63 Eack, S. M., Hogarty, G. E., Greenwald, D. P., Hogarty, S. S., & Keshavan, M. S. (2007). Cognitive enhancement therapy improves emotional intelligence in early course schizophrenia: Preliminary effects. *Schizophrenia Research*, *89*(1–3), 308–311. https://doi.org/10.1016/j.schres.2006.08.018

P64 Eack, S. M., Hogarty, S. S., Greenwald, D. P., Litschge, M. Y., McKnight, S. A. F., Bangalore, S. S., …

Cornelius, J. R. (2015). Cognitive enhancement therapy in substance misusing schizophrenia: Results of an 18-month feasibility trial. *Schizophrenia Research*, *161*(2–3), 478–483. https://doi.org/10.1016/j.schres.2014.11.017

P65 Eack, S. M., Mesholam-Gately, R. I., Greenwald, D. P., Hogarty, S. S., & Keshavan, M. S. (2013). Negative symptom improvement during cognitive rehabilitation: Results from a 2-year trial of Cognitive Enhancement Therapy. *Psychiatry Research*, *209*(1), 21–26. https://doi.org/10.1016/j.psychres.2013.03.020

P66 Eack, S. M., Newhill, C. E., & Keshavan, M. S. (2016). Cognitive enhancement therapy improves resting-state functional connectivity in early course schizophrenia. *Journal of the Society for Social Work and Research*, *7*(2), 211–230. https://doi.org/10.1086/686538

P67 Eack, S. M., Pogue-Geile, M. F., Greenwald, D. P., Hogarty, S. S., & Keshavan, M. S. (2011). Mechanisms of functional improvement in a 2-year trial of cognitive enhancement therapy for early schizophrenia. *Psychological Medicine*, *41*(6), 1253–1261. https://doi.org/10.1017/S0033291710001765

P68 Felisberti, F. M., & Pavey, L. (2010). Contextual modulation of biases in face recognition. *PLoS ONE*, *5*(9), e12939. https://doi.org/10.1371/journal.pone.0012939

P69 Feng, H., Lo, Y. -y., Tsai, S., & Cartledge, G. (2008). The effects of theory-of-mind and social skill training on the social competence of a sixth-grade student with autism. *Journal of Positive Behavior Interventions*, *10*(4), 228–242. https://doi.org/10.1177/1098300708319906

P70 Fernandez-Gonzalo, S., Turon, M., Jodar, M., Pousa, E., Hernandez Rambla, C., García, R., & Palao, D. (2015). A new computerized cognitive and social cognition training specifically designed for patients with schizophrenia/schizoaffective disorder in early stages of illness: A pilot study. *Psychiatry Research*, *228*(3), 501–509. https://doi.org/10.1016/j.psychres.2015.06.007

P71 Fiszdon, J. M., Roberts, D. L., Penn, D. L., Choi, K.-H., Tek, C., Choi, J., & Bell, M. D. (2016). Understanding social situations (USS): A proof-of-concept social–cognitive intervention targeting theory of mind and attributional bias in individuals with psychosis. *Psychiatric Rehabilitation Journal*. https://doi.org/10.1037/prj0000190

P72 Fitzgerald, A., Rawdon, C., & Dooley, B. (2016). A randomized controlled trial of attention bias modification training for socially anxious adolescents. *Behaviour Research and Therapy*, *84*, 1–8. https://doi.org/10.1016/j.brat.2016.06.003

P73 Fu, C. H. Y., Williams, S. C. R., Cleare, A. J., Scott, J., Mitterschiffthaler, M. T., Walsh, N. D., … Murray, R. M. (2008). Neural responses to sad facial expressions in major depression following cognitive behavioral therapy. *Biological Psychiatry*, *64*(6), 505–512. https://doi.org/10.1016/j.biopsych.2008.04.033

P74 Fuentes, I., García, S., Ruiz, J. C., Soler, M. J., & Roder, V. (2007). Social perception training in schizophrenia: A pilot study. *International Journal of Psychology and Psychological Therapy*, *7*(1), 1–12.

P75 Fung, A. (2007). A qualitative evaluation of social-cognitive changes in children with reactively aggressive behaviors. *Journal of School Violence*, *6*(1), 45–64. https://doi.org/10.1300/J202v06n01_04

P76 Gabbatore, I., Sacco, K., Angeleri, R., Zettin, M., Bara, B. G., & Bosco, F. M. (2015). Cognitive pragmatic treatment: A rehabilitative program for traumatic brain injury individuals. *Journal of Head Trauma Rehabilitation*, *30*(5), E14–E28. https://doi.org/10.1097/HTR.0000000000000087

P77 Gabriels, R. L., Pan, Z., Dechant, B., Agnew, J. A., Brim, N., & Mesibov, G. (2015). Randomized controlled trial of therapeutic horseback riding in children and adolescents with autism spectrum disorder. *Journal of the American Academy of Child & Adolescent Psychiatry*, *54*(7), 541–549. https://doi.org/10.1016/j.jaac.2015.04.007

P78 Galende, N., de Miguél, M. S., & Arranz, E. (2012). The role of parents' distancing strategies in the development of five-year-old children's theory of mind. *Early Child Development and Care, 182*(2), 207–220. https://doi.org/10.1080/03004430.2011.553676

P79 Gaudelus, B., Virgile, J., Geliot, S., & Franck, N. (2016). Improving facial emotion recognition in schizophrenia: a controlled study comparing specific and attentional focused cognitive remediation. *Frontiers in Psychiatry*, *7*. https://doi.org/10.3389/fpsyt.2016.00105

P80 Gawęda, Ł., Krężołek, M., Olbryś, J., Turska, A., & Kokoszka, A. (2015). Decreasing self-reported cognitive biases and increasing clinical insight through meta-cognitive training in patients with chronic schizophrenia. *Journal of Behavior Therapy and Experimental Psychiatry*, *48*, 98–104. https://doi.org/10.1016/j.jbtep.2015.02.002

P81 Gevers, C., Clifford, P., Mager, M., & Boer, F. (2006). Brief report: A theory-of-mind-based social-cognition training program for school-aged children with pervasive developmental disorders: An open study of its effectiveness. *Journal of Autism and Developmental Disorders*, *36*(4), 567–571. https://doi.org/10.1007/s10803-006-0095-0

P82 Ghoreishian, M. A., Neshat Doust, H. T., & Molavi, H. (2010). Attributional style retraining and marital satisfaction among Iranian couples. *Developmental Psychology*, *6*(24), 329–336.

P83 Gil-Sanz, D., Fernández-Modamio, M., Bengochea-Seco, R., Arrieta-Rodríguez, M., & Pérez-Fuentes, G. (2014). Efficacy of the social cognition training program in a sample of schizophrenic outpatients. *Clinical Schizophrenia & Related Psychoses*, (aop), 1–27. https://doi.org/10.3371/CSRP.GIFE.013114

P84 Gil-Sanz, D., Fernández-Modamio, M., Bengochea-Seco, R., Arrieta-Rodríguez, M., & Pérez-Fuentes, G. (2016). Efficacy of the social cognition training program in a sample of outpatients with schizophrenia. *Clinical Schizophrenia & Related Psychoses*, *10*(3), 154–162. https://doi.org/10.3371/1935-1232.10.3.154

P85 Gil-Sanz, D., Lorenzo, M. D., Seco, R. B., Rodríguez, M. A., Martínez, I. L., Calleja, R. S., & Soltero, A. Á. (2009). Efficacy of a social cognition training program for schizophrenic patients: A pilot study. *The Spanish Journal of Psychology*, *12*(1), 184–191. https://doi.org/10.1017/S1138741600001591

P86 Gohar, S. M., Hamdi, E., El Ray, L. A., Horan, W. P., & Green, M. F. (2013). Adapting and evaluating a social cognitive remediation program for schizophrenia in Arabic. *Schizophrenia Research*, *148*(1–3), 12–17. https://doi.org/10.1016/j.schres.2013.05.008

P87 Gola, A. A. H. (2012). Mental verb input for promoting children's theory of mind: A training study. *Cognitive Development*, *27*(1), 64–76. https://doi.org/10.1016/j.cogdev.2011.10.003

P88 Golan, O., & Baron-Cohen, S. (2006). Systemizing empathy: Teaching adults with Asperger syndrome or high-functioning autism to recognize complex emotions using interactive multimedia. *Development and Psychopathology*, *18*(2). https://doi.org/10.1017/S0954579406060305

P89 Goldstein, T. R., & Winner, E. (2012). Enhancing empathy and theory of mind. *Journal of Cognition and Development*, *13*(1), 19–37. https://doi.org/10.1080/15248372.2011.573514

P90 Grazzani, I., Ornaghi, V., Agliati, A., & Brazzelli, E. (2016). How to foster toddlers' mental-state talk, emotion understanding, and prosocial behavior: A conversation-based intervention at nursery school. *Infancy*, *21*(2), 199–227. https://doi.org/10.1111/infa.12107

P91 Grazzani, I., Ornaghi, V., & Brockmeier, J. (2016). Conversation on mental states at nursery: Promoting social cognition in early childhood. *European Journal of Developmental Psychology*, *13*(5), 563–581. https://doi.org/10.1080/17405629.2015.1127803

P92 Habel, U., Koch, K., Kellermann, T., Reske, M., Frommann, N., Wölwer, W., … Schneider, F. (2010). Training of affect recognition in schizophrenia: Neurobiological correlates. *Social Neuroscience*, *5*(1), 92–104. https://doi.org/10.1080/17470910903170269

P93 Han, D. H., Chung, U., Shin, Y. J., & Renshaw, P. (2016). A prosocial online game for social cognition training in adolescents with high-functioning autism: an fMRI study. *Neuropsychiatric Disease and Treatment*, 651. https://doi.org/10.2147/NDT.S94669

P94 Hasson-Ohayon, I., Mashiach-Eizenberg, M., Avidan, M., Roberts, D. L., & Roe, D. (2014). Social cognition and interaction training: Preliminary results of an RCT in a community setting in Israel. *Psychiatric Services*, *65*(4), 555–558. https://doi.org/10.1176/appi.ps.201300146

P95 Hawkins, K. A., & Cougle, J. R. (2013). Effects of interpretation training on hostile attribution bias and reactivity to interpersonal insult. *Behavior Therapy*, *44*(3), 479–488. https://doi.org/10.1016/j.beth.2013.04.005

P96 Hernández-Vidal, J. V., Ricarte, J., Ros, L., & Latorre, J. M. (2013). Memoria de trabajo y cambio en la atribución de hostilidad en situaciones sociales accidentales en pacientes con esquizofrenia paranoide. *Revista de Psicología Social*, *28*(3), 361–372. https://doi.org/10.1174/021347413807719120

P97 Herpertz, S., Schütz, A., & Nezlek, J. (2016). Enhancing emotion perception, a fundamental component of emotional intelligence: using multiple-group SEM to evaluate a training program. *Personality and Individual Differences*, *95*, 11–19. https://doi.org/10.1016/j.paid.2016.02.015

P98 Hillis, J. D., Leonhardt, B. L., Vohs, J. L., Buck, K. D., Salvatore, G., Popolo, R., … Lysaker, P. H. (2015). Metacognitive reflective and insight therapy for people in early phase of a schizophrenia spectrum disorder. *Journal of Clinical Psychology*, *71*(2), 125–135. https://doi.org/10.1002/jclp.22148

P99 Hoddenbach, E., Koot, H. M., Clifford, P., Gevers, C., Clauser, C., Boer, F., & Begeer, S. (2012). Individual differences in the efficacy of a short theory of mind intervention for children with autism spectrum disorder: a randomized controlled trial. *Trials*, *13*(1), 206. https://doi.org/10.1186/1745-6215-13-206

P100 Hogarty, G. E., Greenwald, D. P., & Eack, S. M. (2006). Durability and mechanism of effects of cognitive enhancement therapy. *Psychiatric Services*, *57*(12), 1751–1757. https://doi.org/10.1176/appi.ps.57.12.1751

P101 Hooker, C. I., Bruce, L., Fisher, M., Verosky, S. C., Miyakawa, A., D'Esposito, M., & Vinogradov, S. (2013). The influence of combined cognitive plus social-cognitive training on amygdala response during face emotion recognition in schizophrenia. *Psychiatry Research: Neuroimaging*, *213*(2), 99–107. https://doi.org/10.1016/j.pscychresns.2013.04.001

P102 Hooker, C. I., Bruce, L., Fisher, M., Verosky, S. C., Miyakawa, A., & Vinogradov, S. (2012). Neural activity during emotion recognition after combined cognitive plus social cognitive training in schizophrenia. *Schizophrenia Research*, *139*(1–3), 53–59. https://doi.org/10.1016/j.schres.2012.05.009

P103 Horan, W. P., Kern, R. S., Shokat-Fadai, K., Sergi, M. J., Wynn, J. K., & Green, M. F. (2009). Social cognitive skills training in schizophrenia: an initial efficacy study of stabilized outpatients. *Schizophrenia Research*, *107*(1), 47–54. https://doi.org/10.1016/j.schres.2008.09.006

P104 Horan, W. P., Kern, R. S., Tripp, C., Hellemann, G., Wynn, J. K., Bell, M., … Green, M. F. (2011). Efficacy and specificity of social cognitive skills training for outpatients with psychotic disorders. *Journal of Psychiatric Research*, *45*(8), 1113–1122. https://doi.org/10.1016/j.jpsychires.2011.01.015

P105 Iao, L.-S., Leekam, S., Perner, J., & McConachie, H. (2011). Further evidence for nonspecificity of theory of mind in preschoolers: Training and transferability in the understanding of false beliefs and false signs. *Journal of Cognition and Development*, *12*(1), 56–79. https://doi.org/10.1080/15248372.2011.539523

P106 Jarrett, R. B., Vittengl, J. R., Doyle, K., & Clark, L. A. (2007). Changes in cognitive content during and following cognitive therapy for recurrent depression: Substantial and enduring, but not predictive of change in depressive symptoms. *Journal of Consulting and Clinical Psychology*, *75*(3), 432–446. https://doi.org/10.1037/0022-006X.75.3.432

P107 Kandalaft, M. R., Didehbani, N., Krawczyk, D. C., Allen, T. T., & Chapman, S. B. (2013). Virtual reality social cognition training for young adults with high-functioning autism. *Journal of Autism and Developmental Disorders*, *43*(1), 34–44. https://doi.org/10.1007/s10803-012-1544-6

P108 Kayser, N., Sarfati, Y., Besche, C., & Hardy-Baylé, M.-C. (2006). Elaboration of a rehabilitation method based on a pathogenetic hypothesis of “theory of mind” impairment in schizophrenia. *Neuropsychological Rehabilitation*, *16*(1), 83–95. https://doi.org/10.1080/09602010443000236

P109 Keshavan, M. S., Eack, S. M., Wojtalik, J. A., Prasad, K. M. R., Francis, A. N., Bhojraj, T. S., … Hogarty, S. S. (2011). A broad cortical reserve accelerates response to cognitive enhancement therapy in early course schizophrenia. *Schizophrenia Research*, *130*(1–3), 123–129. https://doi.org/10.1016/j.schres.2011.05.001

P110 Khodabakhshi, M., Malekpour, M., & Abedi, A. (2015). To evaluate the effectiveness of the training based on theory of mind on the function of mind reading and the executive functions in the children with autism Spectrum Disorders. *Iranian Journal of Psychiatry and Clinical Psychology*, *21*(2), 155–166.

P111 Kidd, D. C., & Castano, E. (2013). Reading literary fiction improves theory of mind. *Science*, *342*(6156), 377–380. https://doi.org/10.1126/science.1239918

P112 King, A. P., Block, S. R., Sripada, R. K., Rauch, S. A. M., Porter, K. E., Favorite, T. K., … Liberzon, I. (2016). A pilot study of mindfulness-based exposure therapy in OEF/OIF combat veterans with PTSD: altered medial frontal cortex and amygdala responses in social–emotional processing. *Frontiers in Psychiatry*, *7*. https://doi.org/10.3389/fpsyt.2016.00154

P113 Klumpp, H., Fitzgerald, D. A., & Phan, K. L. (2013). Neural predictors and mechanisms of cognitive behavioral therapy on threat processing in social anxiety disorder. *Progress in Neuro-Psychopharmacology and Biological Psychiatry*, *45*, 83–91. https://doi.org/10.1016/j.pnpbp.2013.05.004

P114 Koehne, S., Behrends, A., Fairhurst, M. T., & Dziobek, I. (2016). Fostering social cognition through an imitation- and synchronization-based dance/movement intervention in adults with autism spectrum disorder: a controlled proof-of-concept study. *Psychotherapy and Psychosomatics*, *85*(1), 27–35. https://doi.org/10.1159/000441111

P115 Lacava, P. G., Golan, O., Baron-Cohen, S., & Smith Myles, B. (2007). Using assistive technology to teach emotion recognition to students with asperger syndrome. *Remedial and Special Education*, *28*(3), 174–181. https://doi.org/10.1177/07419325070280030601

P116 Lacava, P. G., Rankin, A., Mahlios, E., Cook, K., & Simpson, R. L. (2010). A single case design evaluation of a software and tutor intervention addressing emotion recognition and social interaction in four boys with ASD. *Autism*, *14*(3), 161–178. https://doi.org/10.1177/1362361310362085

P117 Lahera, G., Benito, A., Montes, J. M., Fernández-Liria, A., Olbert, C. M., & Penn, D. L. (2013). Social cognition and interaction training (SCIT) for outpatients with bipolar disorder. *Journal of Affective Disorders*, *146*(1), 132–136. https://doi.org/10.1016/j.jad.2012.06.032

P118 Lecce, S., Bianco, F., Demicheli, P., & Cavallini, E. (2014). Training preschoolers on first-order false belief understanding: Transfer on advanced ToM skills and metamemory. *Child Development*, n/a-n/a. https://doi.org/10.1111/cdev.12267

P119 Lecce, S., Bianco, F., Devine, R. T., Hughes, C., & Banerjee, R. (2014). Promoting theory of mind during middle childhood: A training program. *Journal of Experimental Child Psychology*, *126*, 52–67. https://doi.org/10.1016/j.jecp.2014.03.002

P120 Lecce, S., Bottiroli, S., Bianco, F., Rosi, A., & Cavallini, E. (2015). Training older adults on theory of mind (ToM): Transfer on metamemory. *Archives of Gerontology and Geriatrics*, *60*(1), 217–226. https://doi.org/10.1016/j.archger.2014.10.001

P121 Lewandowski, K. E., Eack, S. M., Hogarty, S. S., Greenwald, D. P., & Keshavan, M. S. (2011). Is cognitive enhancement therapy equally effective for patients with schizophrenia and schizoaffective disorder? *Schizophrenia Research*, *125*(2–3), 291–294. https://doi.org/10.1016/j.schres.2010.11.017

P122 Lindenmayer, J.-P., McGurk, S. R., Khan, A., Kaushik, S., Thanju, A., Hoffman, L., … Herrmann, E. (2013). Improving social cognition in schizophrenia: A pilot intervention combining computerized social cognition training with cognitive remediation. *Schizophrenia Bulletin*, *39*(3), 507–517. https://doi.org/10.1093/schbul/sbs120

P123 Lopata, C., Thomeer, M. L., Rodgers, J. D., Donnelly, J. P., & McDonald, C. A. (2016). RCT of mind reading as a component of a psychosocial treatment for high-functioning children with ASD. *Research in Autism Spectrum Disorders*, *21*, 25–36. https://doi.org/10.1016/j.rasd.2015.09.003

P124 Lopata, C., Thomeer, M. L., Volker, M. A., Nida, R. E., & Lee, G. K. (2008). Effectiveness of a manualized summer social treatment program for high-functioning children with autism spectrum disorders. *Journal of*

Autism and Developmental Disorders, *38*(5), 890–904. https://doi.org/10.1007/s10803-007-0460-7

P125 Lopata, C., Thomeer, M. L., Volker, M. A., Toomey, J. A., Nida, R. E., Lee, G. K., … Rodgers, J. D. (2010). RCT of a manualized social treatment for high-functioning autism spectrum disorders. *Journal of Autism and Developmental Disorders*, *40*(11), 1297–1310. https://doi.org/10.1007/s10803-010-0989-8

P126 Loukas, K. M., Raymond, L., Perron, A. R., McHarg, L. A., & LaCroix Doe, T. C. (2015). Occupational transformation: Parental influence and social cognition of young adults with autism. *Work*, *50*(3), 457–463. https://doi.org/10.3233/WOR-141956

P127 Lu, H., Su, Y., & Wang, Q. (2008). Talking about others facilitates theory of mind in Chinese preschoolers. *Developmental Psychology*, *44*(6), 1726–1736. https://doi.org/10.1037/a0013074

P128 Luckhaus, C., Frommann, N., Stroth, S., Brinkmeyer, J., & Wölwer, W. (2013). Training of affect recognition in schizophrenia patients with violent offences: Behavioral treatment effects and electrophysiological correlates. *Social Neuroscience*, *8*(5), 505–514. https://doi.org/10.1080/17470919.2013.820667

P129 Lutz, A., Brefczynski-Lewis, J., Johnstone, T., & Davidson, R. J. (2008). Regulation of the neural circuitry of emotion by compassion meditation: Effects of meditative expertise. *PLoS ONE*, *3*(3), e1897. https://doi.org/10.1371/journal.pone.0001897

P130 Marsh, P. J., Langdon, R., McGuire, J., Harris, A., Polito, V., & Coltheart, M. (2013). An open clinical trial assessing a novel training program for social cognitive impairment in schizophrenia. *Australasian Psychiatry*, *21*(2), 122–126. https://doi.org/10.1177/1039856213475683

P131 Marsh, P. J., Green, M. J., Russell, T. A., McGuire, J., Harris, A., & Coltheart, M. (2010). Remediation of facial emotion recognition in schizophrenia: Functional predictors, generalizability, and durability. *American Journal of Psychiatric Rehabilitation*, *13*(2), 143–170. https://doi.org/10.1080/15487761003757066

P132 Marsh, P. J., Luckett, G., Russell, T., Coltheart, M., & Green, M. J. (2012). Effects of facial emotion recognition remediation on visual scanning of novel face stimuli. *Schizophrenia Research*, *141*(2–3), 234–240. https://doi.org/10.1016/j.schres.2012.08.006

P133 Marsh, P. J., Polito, V., Singh, S., Coltheart, M., Langdon, R., & Harris, A. W. (2016). A quasi-randomized feasibility pilot study of specific treatments to improve emotion recognition and mental-state reasoning impairments in schizophrenia. *BMC Psychiatry*, *16*(1), 360. https://doi.org/10.1186/s12888-016-1064-6

P134 Martín García, M. J., Gómez Becerra, I., & Garro Espín, M. J. (2012). Theory of mind in a child with autism: how to train her? *Psicothema*, *24*(4), 542–7. Retrieved from http://www.ncbi.nlm.nih.gov/pubmed/23079349

P135 Mascaro, J. S., Rilling, J. K., Tenzin Negi, L., & Raison, C. L. (2013). Compassion meditation enhances empathic accuracy and related neural activity. *Social Cognitive and Affective Neuroscience*, *8*(1), 48–55. https://doi.org/10.1093/scan/nss095

P136 Mason, L., Peters, E. R., Dima, D., Williams, S. C., & Kumari, V. (2016). Cognitive behavioral therapy normalizes functional connectivity for social threat in psychosis. *Schizophrenia Bulletin*, *42*(3), 684–692. https://doi.org/10.1093/schbul/sbv153

P137 Matsui, M., Arai, H., Yonezawa, M., Sumiyoshi, T., Suzuki, M., & Kurachi, M. (2009). The effects of cognitive rehabilitation on social knowledge in patients with schizophrenia. *Applied Neuropsychology*, *16*(3), 158–164. https://doi.org/10.1080/09084280903098414

P138 Mazza, M., Lucci, G., Pacitti, F., Pino, M. C., Mariano, M., Casacchia, M., & Roncone, R. (2010). Could schizophrenic subjects improve their social cognition abilities only with observation and imitation of social situations? *Neuropsychological Rehabilitation*, *20*(5), 675–703. https://doi.org/10.1080/09602011.2010.486284

P139 Melloni, M., Sedeño, L., Couto, B., Reynoso, M., Gelormini, C., Favaloro, R., … Ibanez, A. (2013). Preliminary evidence about the effects of meditation on interoceptive sensitivity and social cognition. *Behavioral and Brain Functions*, *9*(1), 47. https://doi.org/10.1186/1744-9081-9-47

P140 Mendella, P. D., Burton, C. Z., Tasca, G. A., Roy, P., St. Louis, L., & Twamley, E. W. (2015). Compensatory cognitive training for people with first-episode schizophrenia: Results from a pilot randomized controlled trial. *Schizophrenia Research*, *162*(1–3), 108–111. https://doi.org/10.1016/j.schres.2015.01.016

P141 Money, C., Genders, R., Treasure, J., Schmidt, U., & Tchanturia, K. (2011). A brief emotion focused intervention for inpatients with anorexia nervosa: A qualitative study. *Journal of Health Psychology*, *16*(6), 947–958. https://doi.org/10.1177/1359105310396395

P142 Montag, C., Haase, L., Seidel, D., Bayerl, M., Gallinat, J., Herrmann, U., & Dannecker, K. (2014). A pilot RCT of psychodynamic group art therapy for patients in acute psychotic episodes: Feasibility, impact on symptoms and mentalising capacity. *PLoS ONE*, *9*(11), e112348. https://doi.org/10.1371/journal.pone.0112348

P143 Mueller, D. R., Schmidt, S. J., & Roder, V. (2015). One-year randomized controlled trial and follow-up of integrated neurocognitive therapy for schizophrenia outpatients. *Schizophrenia Bulletin*, *41*(3), 604–616. https://doi.org/10.1093/schbul/sbu223

P144 Nahum, M., Fisher, M., Loewy, R., Poelke, G., Ventura, J., Nuechterlein, K. H., … Vinogradov, S. (2014). A novel, online social cognitive training program for young adults with schizophrenia: A pilot study. *Schizophrenia Research: Cognition*, *1*(1), e11–e19. https://doi.org/10.1016/j.scog.2014.01.003

P145 Neumann, D., Babbage, D. R., Zupan, B., & Willer, B. (2015). A randomized controlled trial of emotion

recognition training after traumatic brain injury. *Journal of Head Trauma Rehabilitation, 30*(3), E12–E23. https://doi.org/10.1097/HTR.0000000000000054

P146 Nikmanesh, Z., Kazemi, Y., Raghibi, M., & Rabani Bavejdan, M. (2012). Effectiveness of optimism skills group training: Examination of the attributional styles of boys at the Kerman juvenile correction and rehabilitation center. *International Journal of High Risk Behaviors and Addiction*, *1*(2), 61–65. https://doi.org/10.5812/ijhrba.4412

P147 Nowakowski, M. E., Antony, M. M., & Koerner, N. (2015). Modifying interpretation biases: Effects on symptomatology, behavior, and physiological reactivity in social anxiety. *Journal of Behavior Therapy and Experimental Psychiatry*, *49*, 44–52. https://doi.org/10.1016/j.jbtep.2015.04.004

P148 Nuechterlein, K. H., Ventura, J., McEwen, S. C., Gretchen-Doorly, D., Vinogradov, S., & Subotnik, K. L. (2016). Enhancing cognitive training through aerobic exercise after a first schizophrenia episode: Theoretical conception and pilot study. *Schizophrenia Bulletin*, *42*(suppl 1), S44–S52. https://doi.org/10.1093/schbul/sbw007

P149 O'Kearney, R., Gibson, M., Christensen, H., & Griffiths, K. M. (2006). Effects of a cognitive-behavioural Internet program on depression, vulnerability to depression and stigma in adolescent males: A school-based controlled trial. *Cognitive Behaviour Therapy*, *35*(1), 43–54. https://doi.org/10.1080/16506070500303456

P150 Ornaghi, V., Brockmeier, J., & Grazzani, I. (2014). Enhancing social cognition by training children in emotion understanding: A primary school study. *Journal of Experimental Child Psychology*, *119*, 26–39. https://doi.org/10.1016/j.jecp.2013.10.005

P151 Ornaghi, V., Grazzani, I., Cherubin, E., Conte, E., & Piralli, F. (2015). "Let"s talk about emotions!'. The effect of conversational training on preschoolers' emotion comprehension and prosocial orientation. *Social Development*, *24*(1), 166–183. https://doi.org/10.1111/sode.12091

P152 Ottavi, P., D'Alia, D., Lysaker, P., Kent, J., Popolo, R., Salvatore, G., & Dimaggio, G. (2014). Metacognition-oriented social skills training for individuals with long-term schizophrenia: Methodology and clinical illustration. *Clinical Psychology & Psychotherapy*, *21*(5), 465–473. https://doi.org/10.1002/cpp.1850

P153 Parker, S., Foley, S., Walker, P., & Dark, F. (2013). Improving the social cognitive deficits of schizophrenia: a community trial of social cognition and interaction training (SCIT). *Australasian Psychiatry*, *21*(4), 346–351. https://doi.org/10.1177/1039856213486305

P154 Parkes, J. F., & Mallett, C. J. (2011). Developing mental toughness: Attributional style retraining in rugby. *The Sport Psychologist, 25*(3), 269–287. https://doi.org/10.1123/tsp.25.3.269

P155 Paynter, J., & Peterson, C. C. (2013). Further evidence of benefits of thought-bubble training for theory of mind development in children with autism spectrum disorders. *Research in Autism Spectrum Disorders*, *7*(2), 344–348. https://doi.org/10.1016/j.rasd.2012.10.001

P156 Penton-Voak, I. S., Bate, H., Lewis, G., & Munafo, M. R. (2012). Effects of emotion perception training on mood in undergraduate students: randomised controlled trial. *The British Journal of Psychiatry*, *201*(1), 71–72. https://doi.org/10.1192/bjp.bp.111.107086

P157 Peña, J., Ibarretxe-Bilbao, N., Garcia-Gorostiaga, I., Gomez-Beldarrain, M. A., Diez-Cirarda, M., & Ojeda, N. (2014). Improving functional disability and cognition in Parkinson disease: Randomized controlled trial. *Neurology*, *83*(23), 2167–2174. https://doi.org/10.1212/WNL.0000000000001043

P158 Peña, J., Ibarretxe-Bilbao, N., Sánchez, P., Iriarte, M. B., Elizagarate, E., Garay, M. A., … Ojeda, N. (2016). Combining social cognitive treatment, cognitive remediation, and functional skills training in schizophrenia: a randomized controlled trial. *NPJ Schizophrenia*, *2*, 16037. https://doi.org/10.1038/npjschz.2016.37

P159 Peña, J., Sánchez, P., Elizagárate, E., Ibarretxe-Bilbao, N., Ezcurra, J., Caballero, L., … Ojeda, N. (2015). Clinical (but not cognitive) recovery in schizophrenia through the experience of fictional cinema. *Schizophrenia Research: Cognition*, *2*(4), 189–194. https://doi.org/10.1016/j.scog.2015.10.003

P160 Peters, K. D., Constans, J. I., & Mathews, A. (2011). Experimental modification of attribution processes. *Journal of Abnormal Psychology*, *120*(1), 168–173. https://doi.org/10.1037/a0021899

P161 Peyroux, E., & Franck, N. (2014). RC2S: A cognitive remediation program to improve social cognition in schizophrenia and related disorders. *Frontiers in Human Neuroscience*, *8*. https://doi.org/10.3389/fnhum.2014.00400

P162 Peyroux, E., & Franck, N. (2016). Improving social cognition in people with schizophrenia with RC2S: Two single-case studies. *Frontiers in Psychiatry*, *7*. https://doi.org/10.3389/fpsyt.2016.00066

P163 Pino, M. C., & Mazza, M. (2016). The use of "literary fiction" to promote mentalizing ability. *PLoS ONE*, *11*(8), e0160254. https://doi.org/10.1371/journal.pone.0160254

P164 Pino, M. C., Pettinelli, M., Clementi, D., Gianfelice, C., & Mazza, M. (2015). Improvement in cognitive and affective theory of mind with observation and imitation treatment in subjects with schizophrenia. *Clinical Neuropsychiatry*, *12*(3), 64–72.

P165 Popova, P., Popov, T. G., Wienbruch, C., Carolus, A. M., Miller, G. A., & Rockstroh, B. S. (2014). Changing facial affect recognition in schizophrenia: Effects of training on brain dynamics. *NeuroImage: Clinical*, *6*, 156–165. https://doi.org/10.1016/j.nicl.2014.08.026

P166 Proudfoot, J. G., Corr, P. J., Guest, D. E., & Dunn, G. (2009). Cognitive-behavioural training to change

attributional style improves employee well-being, job satisfaction, productivity, and turnover. *Personality and Individual Differences*, *46*(2), 147–153. https://doi.org/10.1016/j.paid.2008.09.018

P167 Punamäki, R.-L., Paavonen, J., Toikka, S., & Solantaus, T. (2013). Effectiveness of preventive family intervention in improving cognitive attributions among children of depressed parents: A randomized study. *Journal of Family Psychology*, *27*(4), 683–690. https://doi.org/10.1037/a0033466

P168 Qian, L., Dai, Z., & Zhou, S. (2014). Belief understanding intervention for children with autism. *Chinese Journal of Clinical Psychology*, *3*, 457–461.

P169 Qu, L., Shen, P., Chee, Y. Y., & Chen, L. (2015). Teachers' theory-of-mind coaching and children's executive function predict the training effect of sociodramatic play on children's theory of mind. *Social Development*, *24*(4), 716–733. https://doi.org/10.1111/sode.12116

P170 Quiles, C., Verdoux, H., & Prouteau, A. (2014). Assessing metacognition during a cognitive task: Impact of "on-line" metacognitive questions on neuropsychological performances in a non-clinical sample. *Journal of the International Neuropsychological Society*, *20*(5), 547–554. https://doi.org/10.1017/S1355617714000290

P171 Radice-Neumann, D., Zupan, B., Tomita, M., & Willer, B. (2009). Training emotional processing in persons with brain injury. *Journal of Head Trauma Rehabilitation*, *24*(5), 313–323. https://doi.org/10.1097/HTR.0b013e3181b09160

P172 Rakitzi, S., Georgila, P., Efthimiou, K., & Mueller, D. R. (2016). Efficacy and feasibility of the integrated psychological therapy for outpatients with schizophrenia in Greece: Final results of a RCT. *Psychiatry Research*, *242*, 137–143. https://doi.org/10.1016/j.psychres.2016.05.039

P173 Reboreda, A., Gómez, G. J., Lemos, S., Esteban, E., Oncins, J., & Pereyra, L. (2011). Training emotion recognition program in schizophrenia patients. *Rehabilitacion Psicosocial*, *8*, 8–14.

P174 Reina, R., López, V., Jiménez, M., García-Calvo, T., & Hutzler, Y. (2011). Effects of awareness interventions on children's attitudes toward peers with a visual impairment. *International Journal of Rehabilitation Research*, *34*(3), 243–248. https://doi.org/10.1097/MRR.0b013e3283487f49

P175 Richard, D. A., More, W., & Joy, S. P. (2015). Recognizing emotions: Testing an intervention for children with autism spectrum disorders. *Art Therapy*, *32*(1), 13–19. https://doi.org/10.1080/07421656.2014.994163

P176 Roberts, C. M., Kane, R., Bishop, B., Cross, D., Fenton, J., & Hart, B. (2010). The prevention of anxiety and depression in children from disadvantaged schools. *Behaviour Research and Therapy*, *48*(1), 68–73. https://doi.org/10.1016/j.brat.2009.09.002

P177 Roberts, D. L., Combs, D. R., Willoughby, M., Mintz, J., Gibson, C., Rupp, B., & Penn, D. L. (2014). A randomized, controlled trial of social cognition and interaction training (SCIT) for outpatients with schizophrenia spectrum disorders. *British Journal of Clinical Psychology*, *53*(3), 281–298. https://doi.org/10.1111/bjc.12044

P178 Roberts, D. L., Kleinlein, P., & Stevens, B. (2012). An alternative to generating alternative interpretations in social cognitive therapy for psychosis. *Behavioural and Cognitive Psychotherapy*, *40*(4), 491–495. https://doi.org/10.1017/S1352465812000082

P179 Roberts, D. L., & Penn, D. L. (2009). Social cognition and interaction training (SCIT) for outpatients with schizophrenia: A preliminary study. *Psychiatry Research*, *166*(2–3), 141–147. https://doi.org/10.1016/j.psychres.2008.02.007

P180 Roberts, D. L., Penn, D. L., Labate, D., Margolis, S. A., & Sterne, A. (2010). Transportability and feasibility of social cognition and interaction training (SCIT) in community settings. *Behavioural and Cognitive Psychotherapy*, *38*(1), 35. https://doi.org/10.1017/S1352465809990464

P181 Rocha, N. B. F., & Queirós, C. (2013). Metacognitive and social cognition training (MSCT) in schizophrenia: A preliminary efficacy study. *Schizophrenia Research*, *150*(1), 64–68. https://doi.org/10.1016/j.schres.2013.07.057

P182 Rocha, N. B. F., Queirós, C. L., Bravo, A. R., Silva, A. S. A., Marques, A. P. S., Oliveira, C., … Pereira, N. G. F. (2013). Análise qualitativa do impacto do programa de treino metacognitivo e da cognição social em pessoas com Esquizofrenia. *Estudos de Psicologia (Natal)*, *18*(4), 559–568. https://doi.org/10.1590/S1413-294X2013000400003

P183 Rodgers, J. D., Thomeer, M. L., Lopata, C., Volker, M. A., Lee, G. K., McDonald, C. A., … Biscotto, A. A. (2015). RCT of a psychosocial treatment for children with high-functioning ASD: Supplemental analyses of treatment effects on facial emotion encoding. *Journal of Developmental and Physical Disabilities*, *27*(2), 207–221. https://doi.org/10.1007/s10882-014-9409-x

P184 Rooney, R., Hassan, S., Kane, R., Roberts, C. M., & Nesa, M. (2013). Reducing depression in 9–10 year old children in low SES schools: A longitudinal universal randomized controlled trial. *Behaviour Research and Therapy*, *51*(12), 845–854. https://doi.org/10.1016/j.brat.2013.09.005

P185 Rose, A., Vinogradov, S., Fisher, M., Green, M. F., Ventura, J., Hooker, C., … Nahum, M. (2015). Randomized controlled trial of computer-based treatment of social cognition in schizophrenia: the TRuSST trial protocol. *BMC Psychiatry*, *15*(1), 142. https://doi.org/10.1186/s12888-015-0510-1

P186 Rosi, A., Cavallini, E., Bottiroli, S., Bianco, F., & Lecce, S. (2016). Promoting theory of mind in older adults:

does age play a role? *Aging & Mental Health*, *20*(1), 22–28. https://doi.org/10.1080/13607863.2015.1049118

P187 Rostan, C., Sidera, F., Serrano, J., Amadó, A., Vallès-Majoral, E., Esteban, M., & Serrat, E. (2014). Fostering theory of mind development. Short- and medium-term effects of training false belief understanding. *Infancia Y Aprendizaje*, *37*(3), 498–529. https://doi.org/10.1080/02103702.2014.965464

P188 Russell, T. A., Chu, E., & Phillips, M. L. (2006). A pilot study to investigate the effectiveness of emotion recognition remediation in schizophrenia using the micro-expression training tool. *British Journal of Clinical Psychology*, *45*(4), 579–583. https://doi.org/10.1348/014466505X90866

P189 Russell, T. A., Green, M. J., Simpson, I., & Coltheart, M. (2008). Remediation of facial emotion perception in schizophrenia: Concomitant changes in visual attention. *Schizophrenia Research*, *103*(1–3), 248–256. https://doi.org/10.1016/j.schres.2008.04.033

P190 Russo-Ponsaran, N. M., Evans-Smith, B., Johnson, J., Russo, J., & McKown, C. (2016). Efficacy of a facial emotion training program for children and adolescents with autism spectrum disorders. *Journal of Nonverbal Behavior*, *40*(1), 13–38. https://doi.org/10.1007/s10919-015-0217-5

P191 Sachs, G., Winklbaur, B., Jagsch, R., Lasser, I., Kryspin-Exner, I., Frommann, N., & Wölwer, W. (2012). Training of affect recognition (TAR) in schizophrenia - Impact on functional outcome. *Schizophrenia Research*, *138*(2–3), 262–267. https://doi.org/10.1016/j.schres.2012.03.005

P192 Sacks, S., Fisher, M., Garrett, C., Alexander, P., Holland, C., Rose, D., … Vinogradov, S. (2013). Combining computerized social cognitive training with neuroplasticity-based auditory training in schizophrenia. *Clinical Schizophrenia & Related Psychoses*, *7*(2), 78–86A. https://doi.org/10.3371/CSRP.SAFI.012513

P193 Salgueiro, E., Nunes, L., Barros, A., Maroco, J., Salgueiro, A., & dos Santos, M. E. (2012). Effects of a dolphin interaction program on children with autism spectrum disorders – an exploratory research. *BMC Research Notes*, *5*(1), 199. https://doi.org/10.1186/1756-0500-5-199

P194 Sanefuji, W., & Ohgami, H. (2013). "Being-imitated" strategy at home-based intervention for young children with autism. *Infant Mental Health Journal*, *34*(1), 72–79. https://doi.org/10.1002/imhj.21375

P195 Santiesteban, I., White, S., Cook, J., Gilbert, S. J., Heyes, C., & Bird, G. (2012). Training social cognition: From imitation to theory of mind. *Cognition*, *122*(2), 228–235. https://doi.org/10.1016/j.cognition.2011.11.004

P196 Schönenberg, M., Christian, S., Gaußer, A.-K., Mayer, S. V., Hautzinger, M., & Jusyte, A. (2014). Addressing perceptual insensitivity to facial affect in violent offenders: first evidence for the efficacy of a novel implicit training approach. *Psychological Medicine*, *44*(5), 1043–1052. https://doi.org/10.1017/S0033291713001517

P197 Serrat Sellabona, E., Serrano Ortiz, J., Amadó Codony, A., Sidera Caballero, F., Andrés Roqueta, C., Lloveras, S., & Badia, I. (2012). Entrenar la comprensión de la falsa creencia en niños con alteraciones del lenguaje. *Revista de Logopedia, Foniatría Y Audiología*, *32*(3), 109–119. https://doi.org/10.1016/j.rlfa.2012.01.003

P198 Serret, S., Hun, S., Iakimova, G., Lozada, J., Anastassova, M., Santos, A., … Askenazy, F. (2014). Facing the challenge of teaching emotions to individuals with low- and high-functioning autism using a new serious game: A pilot study. *Molecular Autism*, *5*(1), 37. https://doi.org/10.1186/2040-2392-5-37

P199 Shashi, V., Harrell, W., Eack, S., Sanders, C., McConkie-Rosell, A., Keshavan, M. S., … Hooper, S. R. (2015). Social cognitive training in adolescents with chromosome 22q11.2 deletion syndrome: feasibility and preliminary effects of the intervention. *Journal of Intellectual Disability Research*, *59*(10), 902–913. https://doi.org/10.1111/jir.12192

P200 Soleymani, M., Mohammad, K. P., & Doulatshari, B. (2008). Effectiveness of brief group interpersonal psychotherapy in reducing university students' depressive symptoms and its effect on attributional style and dysfunctional attitudes. *Psychological Research*, *11*(1–2), 41–65.

P201 Soorya, L. V., Siper, P. M., Beck, T., Soffes, S., Halpern, D., Gorenstein, M., … Wang, A. T. (2015). Randomized comparative trial of a social cognitive skills group for children with autism spectrum disorder. *Journal of the American Academy of Child & Adolescent Psychiatry*, *54*(3), 208–216.e1. https://doi.org/10.1016/j.jaac.2014.12.005

P202 Souto, T., Baptista, A., Tavares, D., Queirós, C., & Marques, A. (2013). Facial emotional recognition in schizophrenia: Preliminary results of the virtual reality program for facial emotional recognition. *Revista de Psiquiatria Clínica*, *40*(4), 129–134. https://doi.org/10.1590/S0101-60832013000400001

P203 Sportel, B. E., de Hullu, E., de Jong, P. J., & Nauta, M. H. (2013). Cognitive bias modification versus CBT in reducing adolescent socialanxiety: A randomized controlled trial. *PLoS ONE*, *8*(5), e64355. https://doi.org/10.1371/journal.pone.0064355

P204 Stroth, S., Kamp, D., Drusch, K., Frommann, N., & Wölwer, W. (2015). Training of affect recognition impacts electrophysiological correlates of facial affect recognition in schizophrenia: Analyses of fixation-locked potentials. *The World Journal of Biological Psychiatry*, *16*(6), 411–421. https://doi.org/10.3109/15622975.2015.1051110

P205 Stueck, M., Villegas, A., Lahn, F., Bauer, K., Tofts, P., & Sack, U. (2016). Biodanza for kindergarten children (TANZPRO-Biodanza): reporting on changes of cortisol levels and emotion recognition. *Body, Movement and Dance in Psychotherapy*, *11*(1), 75–89. https://doi.org/10.1080/17432979.2015.1124923

P206 Taksal, A., Sudhir, P. M., Janakiprasad, K. K., Viswanath, D., & Thirthalli, J. (2016). Impact of the integrated psychological treatment (IPT) on social cognition, social skills and functioning in persons diagnosed with

schizophrenia: A feasibility study from India. *Psychosis*, *8*(3), 214–225. https://doi.org/10.1080/17522439.2015.1088058

P207 Tan, L. B. G., Lo, B. C. Y., & Macrae, C. N. (2014). Brief mindfulness meditation improves mental state attribution and empathizing. *PLoS ONE*, *9*(10), e110510. https://doi.org/10.1371/journal.pone.0110510

P208 Tardif, C., Lainé, F., Rodriguez, M., & Gepner, B. (2007). Slowing down presentation of facial movements and vocal sounds enhances facial expression recognition and induces facial–vocal imitation in children with autism. *Journal of Autism and Developmental Disorders*, *37*(8), 1469–1484. https://doi.org/10.1007/s10803-006-0223-x

P209 Tas, C., Danaci, A. E., Cubukcuoglu, Z., & Brüne, M. (2012). Impact of family involvement on social cognition training in clinically stable outpatients with schizophrenia - A randomized pilot study. *Psychiatry Research*, *195*(1–2), 32–38. https://doi.org/10.1016/j.psychres.2011.07.031

P210 Taumoepeau, M., & Reese, E. (2013). Maternal reminiscing, elaborative talk, and children's theory of mind: An intervention study. *First Language*, *33*(4), 388–410. https://doi.org/10.1177/0142723713493347

P211 Taylor, R., Cella, M., Csipke, E., Heriot-Maitland, C., Gibbs, C., & Wykes, T. (2016). Tackling social cognition in schizophrenia: A randomized feasibility trial. *Behavioural and Cognitive Psychotherapy*, *44*(3), 306–317. https://doi.org/10.1017/S1352465815000284

P212 Tchanturia, K., Doris, E., Mountford, V., & Fleming, C. (2015). Cognitive remediation and emotion skills training (CREST) for anorexia nervosa in individual format: self-reported outcomes. *BMC Psychiatry*, *15*(1), 53. https://doi.org/10.1186/s12888-015-0434-9

P213 Thomeer, M. L., Lopata, C., Donnelly, J. P., Booth, A., Shanahan, A., Federiconi, V., … Rodgers, J. D. (2016). Community effectiveness RCT of a comprehensive psychosocial treatment for high-functioning children with ASD. *Journal of Clinical Child & Adolescent Psychology*, 1–12. https://doi.org/10.1080/15374416.2016.1247359

P214 Thomeer, M. L., Lopata, C., Volker, M. A., Toomey, J. A., Lee, G. K., Smerbeck, A. M., … Smith, R. A. (2012). Randomized clinical trial replication of a psychosocial treatment for children with high-functioning autism spectrum disorders. *Psychology in the Schools*, *49*(10), 942–954. https://doi.org/10.1002/pits.21647

P215 Thomeer, M. L., Rodgers, J. D., Lopata, C., McDonald, C. A., Volker, M. A., Toomey, J. A., … Gullo, G. (2011). Open-trial pilot of mind reading and in vivo rehearsal for children with HFASD. *Focus on Autism and Other Developmental Disabilities*, *26*(3), 153–161. https://doi.org/10.1177/1088357611414876

P216 Thomeer, M. L., Smith, R. A., Lopata, C., Volker, M. A., Lipinski, A. M., Rodgers, J. D., … Lee, G. K. (2015). Randomized controlled trial of mind reading and in vivo rehearsal for high-functioning children with ASD. *Journal of Autism and Developmental Disorders*, *45*(7), 2115–2127. https://doi.org/10.1007/s10803-015-2374-0

P217 Tompkins, V. (2015). Improving low-income preschoolers' theory of mind: A training study. *Cognitive Development*, *36*, 1–19. https://doi.org/10.1016/j.cogdev.2015.07.001

P218 Tucci, S. L., Easterbrooks, S. R., & Lederberg, A. R. (2016). The effects of theory of mind training on the false belief understanding of deaf and hard-of-hearing students in prekindergarten and kindergarten. *Journal of Deaf Studies and Deaf Education*, *21*(3), 310–325. https://doi.org/10.1093/deafed/enw031

P219 Turner-Brown, L. M., Perry, T. D., Dichter, G. S., Bodfish, J. W., & Penn, D. L. (2008). Brief report: Feasibility of social cognition and interaction training for adults with high functioning autism. *Journal of Autism and Developmental Disorders*, *38*(9), 1777–1784. https://doi.org/10.1007/s10803-008-0545-y

P220 Ussorio, D., Giusti, L., Wittekind, C. E., Bianchini, V., Malavolta, M., Pollice, R., … Roncone, R. (2016). Metacognitive training for young subjects (MCT young version) in the early stages of psychosis: Is the duration of untreated psychosis a limiting factor? *Psychology and Psychotherapy: Theory, Research and Practice*, *89*(1), 50–65. https://doi.org/10.1111/papt.12059

P221 Valle, A., Massaro, D., Castelli, I., Sangiuliano Intra, F., Lombardi, E., Bracaglia, E., & Marchetti, A. (2016). Promoting mentalizing in pupils by acting on teachers: Preliminary Italian evidence of the "Thought in Mind" project. *Frontiers in Psychology*, *7*. https://doi.org/10.3389/fpsyg.2016.01213

P222 Vassilopoulos, S. P., Brouzos, A., & Andreou, E. (2015). A multi-session attribution modification program for children with aggressive behaviour: Changes in attributions, emotional reaction estimates, and self-reported aggression. *Behavioural and Cognitive Psychotherapy*, *43*(5), 538–548. https://doi.org/10.1017/S1352465814000149

P223 Vassilopoulos, S. P., Brouzos, A., Damer, D. E., Mellou, A., & Mitropoulou, A. (2013). A psychoeducational school-based group intervention for socially anxious children. *The Journal for Specialists in Group Work*, *38*(4), 307–329. https://doi.org/10.1080/01933922.2013.819953

P224 Vassilopoulos, S. P., Moberly, N. J., & Zisimatou, G. (2013). Experimentally modifying interpretations for positive and negative social scenarios in children: A preliminary investigation. *Behavioural and Cognitive Psychotherapy*, *41*(1), 103–116. https://doi.org/10.1017/S1352465812000537

P225 Vázquez-Campo, M., Maroño, Y., Lahera, G., Mateos, R., & García-Caballero, A. (2016). e-Motional Training®: Pilot study on a novel online training program on social cognition for patients with schizophrenia. *Schizophrenia Research: Cognition*, *4*, 10–17. https://doi.org/10.1016/j.scog.2015.11.007

P226 Velligan, D. I., Roberts, D., Mintz, J., Maples, N., Li, X., Medellin, E., & Brown, M. (2015). A randomized pilot study of MOtiVation and Enhancement (MOVE) Training for negative symptoms in schizophrenia. *Schizophrenia Research*, *165*(2–3), 175–180. https://doi.org/10.1016/j.schres.2015.04.008

P227 Veltro, F., Mazza, M., Vendittelli, N., Alberti, M., Casacchia, M., & Roncone, R. (2011). A comparison of the effectiveness of problem solving training and of cognitive-emotional rehabilitation on neurocognition, social cognition and social functioning in people with schizophrenia. *Clinical Practice & Epidemiology in Mental Health*, *7*(1), 123–132. https://doi.org/10.2174/1745017901107010123

P228 Voutilainen, G., Kouhia, T., Roberts, D. L., & Oksanen, J. (2016). Social cognition and interaction training (SCIT) for adults with psychotic disorders: A feasibility study in Finland. *Behavioural and Cognitive Psychotherapy*, *44*(6), 711–716. https://doi.org/10.1017/S1352465816000151

P229 Wain, H., Kneebone, I. I., & Cropley, M. (2011). Attributional intervention for depression in two people with multiple sclerosis (MS): Single case design. *Behavioural and Cognitive Psychotherapy*, *39*(1), 115–121. https://doi.org/10.1017/S1352465810000536

P230 Wang, Y., Roberts, D. L., Xu, B., Cao, R., Yan, M., & Jiang, Q. (2013). Social cognition and interaction training for patients with stable schizophrenia in Chinese community settings. *Psychiatry Research*, *210*(3), 751–755. https://doi.org/10.1016/j.psychres.2013.08.038

P231 Ware, A., Wilson, C., Tapp, J., & Moore, E. (2016). Mentalisation-based therapy (MBT) in a high-secure hospital setting: expert by experience feedback on participation. *The Journal of Forensic Psychiatry & Psychology*, *27*(5), 722–744. https://doi.org/10.1080/14789949.2016.1174725

P232 Warrender, D. (2015). Staff nurse perceptions of the impact of mentalization-based therapy skills training when working with borderline personality disorder in acute mental health: a qualitative study. *Journal of Psychiatric and Mental Health Nursing*, *22*(8), 623–633. https://doi.org/10.1111/jpm.12248

P233 Weinger, P. M., & Depue, R. A. (2011). Remediation of deficits in recognition of facial emotions in children with autism spectrum disorders. *Child & Family Behavior Therapy*, *33*(1), 20–31. https://doi.org/10.1080/07317107.2011.545008

P234 Wellman, H. M., & Peterson, C. C. (2013). Deafness, thought bubbles, and theory-of-mind development. *Developmental Psychology*, *49*(12), 2357–2367. https://doi.org/10.1037/a0032419

P235 Williams, B. T., Gray, K. M., & Tonge, B. J. (2012). Teaching emotion recognition skills to young children with autism: a randomised controlled trial of an emotion training programme. *Journal of Child Psychology and Psychiatry*, *53*(12), 1268–1276. https://doi.org/10.1111/j.1469-7610.2012.02593.x

P236 Williamson, J., & Isaki, E. (2015). Facial affect recognition training through telepractice: Two case studies of individuals with chronic traumatic brain injury. *International Journal of Telerehabilitation*, *7*(1), 13–20. https://doi.org/10.5195/IJT.2015.6167

P237 Wölwer, W., & Frommann, N. (2008). Therapy of social-cognitive impairments - Training of affect recognition as functional specific approach. *Nervenheilkunde*, *27*(11), 1032–1038.

P238 Wölwer, W., & Frommann, N. (2011). Social-cognitive remediation in schizophrenia: Generalization of effects of the training of affect recognition (TAR). *Schizophrenia Bulletin*, *37*(suppl 2), S63–S70. https://doi.org/10.1093/schbul/sbr071

P239 Xiao, X., Yang, N., Qian, L., & Zhou, S. (2014). Pretend playing training improves theory of mind in children with autism. *Chinese Journal of Clinical Psychology*, *22*(4), 742–745.

P240 Zhang, D.-K., Xu, S.-Q., Su, Q.-R., Pan, Y.-N., Shen, X.-Y., Chen, S.-M., … Shan, X.-Y. (2012). Effects of cognitive and skilled rehabilitative training on deficiencies of theory of mind in patients with traumatic brain injury: A 12-week random, single-blind clinical trial. *Chinese Mental Health Journal*, *12*, 906–912.

P241 Zimmer, M., Duncan, A. V., Laitano, D., Ferreira, E. E., & Belmonte-de-Abreu, P. (2007). A twelve-week randomized controlled study of the cognitive-behavioral Integrated Psychological Therapy program: positive effect on the social functioning of schizophrenic patients. *Revista Brasileira de Psiquiatria*, *29*(2), 140–7. Retrieved from http://www.ncbi.nlm.nih.gov/pubmed/17650536
